# Coresets for the Average Case Error for Finite Query Sets

**DOI:** 10.3390/s21196689

**Published:** 2021-10-08

**Authors:** Alaa Maalouf, Ibrahim Jubran, Murad Tukan, Dan Feldman

**Affiliations:** Robotics & Big Data Labs, University of Haifa, Haifa 3498838, Israel; ijubran@staff.haifa.ac.il (I.J.); mtukan@campus.haifa.ac.il (M.T.); dfeldman@univ.haifa.ac.il (D.F.)

**Keywords:** coreset, average case analysis, big data, sparsification, dimensionality reduction, approximation algorithms

## Abstract

Coreset is usually a small weighted subset of an input set of items, that provably approximates their loss function for a given set of queries (models, classifiers, hypothesis). That is, the maximum (worst-case) error over all queries is bounded. To obtain smaller coresets, we suggest a natural relaxation: coresets whose average error over the given set of queries is bounded. We provide both deterministic and randomized (generic) algorithms for computing such a coreset for any finite set of queries. Unlike most corresponding coresets for the worst-case error, the size of the coreset in this work is independent of both the input size and its Vapnik–Chervonenkis (VC) dimension. The main technique is to reduce the average-case coreset into the vector summarization problem, where the goal is to compute a weighted subset of the *n* input vectors which approximates their sum. We then suggest the first algorithm for computing this weighted subset in time that is linear in the input size, for n≫1/ε, where ε is the approximation error, improving, e.g., both [ICML’17] and applications for principal component analysis (PCA) [NIPS’16]. Experimental results show significant and consistent improvement also in practice. Open source code is provided.

## 1. Introduction

In this paper, we assume that the input is a set *P* of items, called points. Usually, *P* is simply a finite set of *n* points in $R^{d}$ or other metric space. In the context of PAC (probably approximately correct) learning [1], or empirical risk minimization [2] it represents the training set. In supervised learning every point in *P* may also include its label or class. We also assume a given function w:P→(0,∞) called weights function that assigns a “weight” w(p)>0 for every point p∈P. The weights function represents a distribution of importance over the input points, where the natural choice is uniform distribution, i.e., w(p)=1/|P| for every p∈P. We are also given a (possibly infinite) set *X* that is the set of queries [3] which represents candidate models or hypothesis, e.g., neural networks [4], SVMs [5] or a set of vectors in $R^{d}$ with tuning parameters as in linear/ridge/lasso regression [6,7,8].

In machine learning and PAC-learning in particular, we often seek to compute the query that best describes our input data *P* for either prediction, classification, or clustering tasks. To this end, we define a loss functionf:P×X→R that assigns a fitting cost f(p,x) to every point p∈P with respect to a query x∈X. For example, it may be a kernel function [9], a convex function [10], or an inner product. The tuple (P,w,X,f) is called a query space and represents the input to our problem. In this paper, we wish to approximate the weighted sum of losses $f_{w}\left(P,x\right)=\sum_{p\in P}w\left(p\right)f\left(p,x\right)$.

Methodology.

Our main tool for approximating the sum of losses above is called a coreset, which is a (small) weighted subset of the (potentially huge) input data, from which the desired sum of losses can be recovered in a very fast time, with guarantees on the small induced error; see Section 1.2. To compute such a coreset, we utilize the famous Frank–Wolfe algorithm. However, to compute a coreset in fast time, we first provide a scheme for provably boosting the running time of the Frank–Wolfe algorithm for our special case, without compromising its output accuracy; see Section 3.1. We then utilize the boosted version in order to compute a deterministic coreset in time faster than the state of the art.

### 1.1. Approximation Techniques for the Sum of Losses

**Approximating the loss of a single query via uniform sampling.** Suppose that we wish to approximate the mean $f\left(P,x\right)=\frac{1}{n}\sum_{p\in P}f\left(p,x\right)$ for a specific *x* in sub-linear time. Picking a random point *p* uniformly at random from *P* would give this result in expectation as $E\left[f\left(p,x\right)\right]=\sum_{p\in P}f\left(p,x\right)/n=f\left(P,x\right)$. By Hoeffding inequality, the mean $f\left(S,x\right)=\frac{1}{\left|S\right|}\sum_{p\in S}f\left(p,x\right)$ of a uniform sample S⊆P would approximate f(P,x) with high probability. More precisely, for a given ε∈(0,1), if the size of the sample is $\left|S|\in O(M/\varepsilon^{2}\right)$ where $M=\max_{p\in P}\left|f\left(p,x\right)\right|$ is the maximum absolute value of *f*, then with constant probability our approximation error is

$$err_{f}\left(x\right)=\left|f\left(P,x\right)-f\left(S,x\right)\right|\leq \varepsilon .$$

**ε-Sample.** Generally, we are interested in such data summarization *S* of *P* that approximates every query x∈X. An *ε*-sample is a pair (S,u) where *S* is a subset of *P* (unlike, e.g., sketches [11]), and u:S→[0,∞) is its weights function such that the weighted loss $f_{u}\left(S,x\right)=$$\sum_{p\in S}u\left(p\right)f\left(p,x\right)$ of the (hopefully small) weighted subset *S* approximates the original weighted loss $f_{w}\left(P,x\right)$ [12], i.e.,

(1) $$\forall x\in X:\left|f_{w}\left(P,x\right)-f_{u}\left(S,x\right)\right|\leq \varepsilon .$$

We usually assume that the input is normalized in the sense that *w* is a distribution, and f:P×X→[−1,1]. By defining the pair of vectors $f_{w}\left(P,X\right)={\left(\sum_{p\in P}w\left(p\right)f\left(p,x\right)\right)}_{x\in X}$ and $f_{u}\left(S,X\right)={\left(\sum_{p\in S}u\left(p\right)f\left(p,x\right)\right)}_{x\in X}$, we can define the error for a single *x* by $err\left(x\right)=|f_{w}\left(P,x\right)-f_{u}\left(S,x\right)|$, and then the error vector for the coreset $err\left(X\right)={\left(err\left(x\right)\right)}_{x\in X}$. We can rewrite (1) by

(2) $${∥err(X)∥}_{\infty }={∥f_{w}\left(P,X\right)-f_{u}\left(S,X\right)∥}_{\infty }\leq \varepsilon .$$

**PAC/DAC learning for approximating the sum of losses for multiple queries.** Probably approximately correct (PAC) randomized constructions generalizes the Hoeffding inequality above from a single to multiple (usually infinite) queries and returns an ε-sample for a given query space (P,w,X,f) and δ∈(0,1), with probability at least 1−δ. Here, δ corresponds to the “probably” part, while “approximately correct” corresponds to ε in (2); see  [13,14]. Deterministic approximately correct (DAC) versions of PAC-learning suggest deterministic construction of ε-samples, i.e., the probability of failure of the construction is δ=0.

As common in machine learning and computer science in general, the main advantage of deterministic constructions is smaller bounds (in this case, on the size of the resulting ε-sample), and their disadvantage is usually the slower construction time that may be unavoidable. When the query set *X* if finite, the Caratheodory theorem [15,16] suggests a deterministic algorithm that returns a 0-sample (S,u) (i.e., $f_{w}\left(P,X\right)=f_{u}\left(S,X\right)$) of size |S|≤|X|+1. Deterministic constructions of ε-sample are known for infinite sets of queries even when the VC-dimension is unbounded [17,18].

**Sup-sampling: reducing the sample size via non-uniform sampling.** As explained above, Hoeffding inequality implies an approximation of $f_{w}\left(P,x\right)$ by $f_{u}\left(S,x\right)$ where u(p)=1/|S| and *S* is a random sample according to *w* whose size depends on $M\left(f\right)=\max_{p\in P}\left|f\left(p,x\right)\right|$. To reduce the sample size we may thus define $g\left(p,x\right)=\frac{f(p,x)}{\left|f(p,x)\right|}\in \left\{-1,1\right\}$, and $s\left(p\right)=\frac{w(p)|f(p,x)|}{\sum_{q\in P}w\left(q\right)\left|f\left(q,x\right)\right|}$. Now, $M\left(g\right)=\max_{p\in P}\left|g\left(p,x\right)\right|=1$, and by Hoeffding’s inequality, the error of approximating $g_{s}\left(P,x\right)$ via non-uniform random sample of size $1/\varepsilon^{2}$ drawn from *s*, is ε. Define $T=\sum_{q\in P}w\left(q\right)\left|f\left(q,x\right)\right|$. Since $f_{w}\left(P,x\right)=T\cdot g_{s}\left(P,x\right)$, approximating $g_{s}\left(P,x\right)$ up to ε error yields an error of εT for $f_{w}\left(p,x\right)$. Therefore, the size is reduced from $M\left(f\right)/\varepsilon^{2}$ to $T^{2}/\varepsilon^{2}$ when $T^{2}\leq M\left(f\right)$. Here, we sample $\left|S|=O(T^{2}/\varepsilon^{2}\right)$ points from *P* according to the distribution *s*, and re-weight the sampled points by $u^{\prime }\left(p\right)=\frac{T}{\left|S||f(p,x)\right|}$.

Unlike traditional PAC-learning, the sample now is non-uniform, and is proportional to s(p), rather than *w*, as implied by Hoeffding inequality for non-uniform distributions. For sets of queries we generalize the definition for every p∈P to $s\left(p\right)=\sup_{x\in X}\frac{w(p)|f(p,x)|}{\sum_{q\in P}w\left(q\right)\left|f\left(p,x\right)\right|}$ as in [19], which is especially useful for coresets below.

### 1.2. Coresets: A Data Summarization Technique for Approximating the Sum of Losses

Coreset for a given query space (P,w,X,g), in this and many other papers, is a pair (C,u) that is similar to ε-sample in the sense that C⊆P and u:C→[0,∞) is a weights function. However, the additive error ε is now replaced with a multiplicative error 1±ε, i.e., for every x∈X we require that, $|g_{w}\left(P,X\right)-g_{u}\left(C,X\right)|\leq \varepsilon \cdot g_{w}\left(P,X\right).$ Dividing by $g_{w}\left(P,X\right)$ and assuming $g_{w}\left(P,X\right)>0$, yields

(3) $$\forall x\in X:|1-\frac{g_{u}\left(C,X\right)}{g_{w}\left(P,x\right)}|\leq \varepsilon .$$

Coresets are especially useful for learning big data since an off-line and possibly inefficient coreset construction for “small data" implies constructions that maintains coreset for streaming, dynamic (including deletions) and distributed data in parallel. This is via a simple and easy to implement framework that is sometimes called merge–reduce trees; see [20,21]. The fact that a coreset approximates every query (and not just the optimal one for some criterion) implies that we may solve hard optimization problems with non-trivial and non-convex constraints by running a possibly inefficient algorithm such as exhaustive search on the coreset, or running existing heuristics numerous times on the small coreset instead of once on the original data. Similarly, parameter tuning or cross validation can be applied on a coreset that is computed once for the original data as explained in [22].

An ε-coreset for a query space (P,w,X,g) is simply an ε-sample for the query space (P,w,X,f), after defining $f\left(p,x\right):=\frac{g(p,x)}{g_{w}\left(P,x\right)}$, as explained, e.g., in [19]. By defining the error for a single *x* by $err^{\prime }\left(x\right)=|1-g_{u}\left(C,x\right)/g_{w}\left(P,x\right)\left|=\right|f_{w}\left(P,x\right)-f_{u}\left(C,x\right)|=err_{f}\left(x\right)$, we obtain an error vector for the coreset $err^{\prime }\left(X\right)={\left(err^{\prime }\left(x\right)\right)}_{x\in X}$. We can then rewrite (3) as in (2):

$${∥err^{\prime }\left(X\right)∥}_{\infty }\leq \varepsilon .$$

In the case of coresets, the $\underset{x\in X}{\sup}w\left(p\right)f\left(p,x\right)=\underset{x\in X}{\sup}\frac{w(p)g(p,x)}{g_{w}\left(P,x\right)}$ of a point p∈P is called sensitivity [14], leverage score (in $\ell_{2}$ approximations) [23], Lewis weights (in $\ell_{p}$ approximations), or simply importance [24].

### 1.3. Problem Statement: Average Case Analysis for Data Summarization

Average case analysis (e.g., [25]) was suggested about a decade ago as an alternative to the (sometimes infamous) worst-case analysis of algorithms in theoretical computer science. The idea is to replace the analysis for the worst-case input by the average input (in some sense). Inspired by this idea, a natural variant of (2) and its above implications is an ε-sample that approximates well the average query. We suggest to define an $\left(\varepsilon ,∥\cdot ∥\right)$-sample as

(4) $$∥err(X)∥=∥f_{w}\left(P,X\right)-f_{u}\left(S,X\right)∥\leq \varepsilon ,$$

which generalizes (2) from ${∥\cdot ∥}_{\infty }$ to any norm, such as the $\ell_{z}$ norm ${∥err(X)∥}_{z}$. For example, for the $\ell_{2}$, MSE or Frobenius norm, we obtain

(5) $$\begin{array}{c} \sqrt{\sum_{x\in X}{\left(f_{w}\left(P,x\right)-f_{u}\left(S,x\right)\right)}^{2}}\leq \varepsilon . \end{array}$$

A generalization of the Hoeffding Inequality from 1963 with tight bounds was suggested relatively recently for the $\ell_{z}$ norm for any z≥2 and many other norms [26,27]. Here we assume a single query (|X|=1), a distribution weights function, and a bound on $\sup_{p\in P}\left|f(p,X)\right|$ that determines the size of the sample, as in Hoeffding inequality.

A less obvious question, which is the subject of this paper, is how to compute deterministic ε-samples that satisfies (4), for norms other than the infinity norm. While the Caratheodory theorem suggests deterministic constructions of 0-samples (for any error norm) as explained above, our goal is to obtain coreset whose size is smaller or independent of |X|.

The next question is how to generalize the idea of sup-sampling, i.e., where the function *f* is unbounded, for the case of norms other than ${∥\cdot ∥}_{\infty }$. Our main motivation for doing so is to obtain new and smaller coresets by combining the notion of ε-sample and sup-sampling or sensitivity as explained above for the ${∥\cdot ∥}_{\infty }$ case. That is, we wish a coreset for a given query space, that would bound the non-$\ell_{\infty }$ norm error

$$∥{\left(1-\frac{g_{u}\left(C,x\right)}{g_{w}\left(P,x\right)}\right)}_{x\in X}∥=∥err^{\prime }\left(X\right)∥\leq \varepsilon .$$

To summarize, our questions are: **How can we smooth the error function and approximate the “average” query via: (i) Deterministic ε-samples (for DAC-learning )? (ii) Coresets (via sensitivities/sup sampling for non-infinity norms)?**

### 1.4. Our Contribution

We answer affirmably these questions by suggesting ε-samples and coresets for the average query. We focus on the case z=2, i.e., the Frobenius norm, and finite query set *X* and hope that this would inspire the research and applications of other norms and general sets. For suggestions in this direction and future work see Section 5.2. The main results of this paper are the following constructions of an $\left(\varepsilon ,{∥\cdot ∥}_{2}\right)$-sample (S,u) for any given finite query space (P,w,X,f) as defined in (5):

(i) Deterministic construction that returns a coreset of size $\left|S|\in O(1/\varepsilon^{2}\right)$ in time $O\left(\min\left\{nd/\varepsilon^{2},nd+d\log{\left(n\right)}^{2}/\varepsilon^{4}\right\}\right)$; see Theorem 2 and Corollary 4.
(ii) Randomized construction that returns such a coreset (of size $\left|S|\in O(1/\varepsilon^{2}\right)$) with probability at least 1−δ in sub-linear time $O\left(d\left(\log{\left(\frac{1}{\delta }\right)}^{2}+\frac{\log\left(\frac{1}{\delta }\right)}{\varepsilon^{2}}\right)\right)$; see Lemma 5.

Algorithm. This result is of independent interest for faster and sparser convex optimization. To our knowledge, this is also the first application of sensitivity outside the coreset regime.

### 1.5. Overview and Organization

The rest of the paper in organized as follows. First in Section 2, we list the applications of our proposed methods, such as a faster coreset construction algorithm for least mean squares solver. We also compare our results to the state of the art to justify our practical contribution.

In Section 3, we first give our notations and relevant mathematical definitions, we explain the relation between the problem of computing an $\left(\varepsilon ,{∥\cdot ∥}_{2}\right)$-sample (average-case coreset) to the problem of computing a vector summarization coreset, where the goal (of the vector summarization coreset problem) is to compute a weighted subset of the *n* input vectors which approximates their sum. Here, we suggest a coreset for this problem of size O(1/ε) in O(nd/ε) time; see Theorem 2 and Algorithm 2. Then, in Section 3.1 we show how to improve the running time of this result and compute a coreset of the same size in $O(nd+d\log^{2}\left(n\right)/\varepsilon^{2})$ time; see Corollary 4 and Algorithm 3. In addition, we suggest a non-deterministic coreset of the same size but in time that is independent of the number of points *n*; see Lemma 5 and Algorithm 4.

In Section 4, we explain how our vector summarization coreset results can be used to improve all the previously mentioned applications (from Section 2). In Section 5 we conduct various experiments on real world datasets, where we apply different coreset construction algorithms presented in this paper to a variety of applications, in order to boost their running time, or reduce their memory storage. We also compare our results to many competing methods. Finally, we conclude our paper and discuss future work at Section 5.2. Due to space limitations and simplicity of the reading, the proofs of the claims are placed in the Appendix A, Appendix B, Appendix C, Appendix D, Appendix E, Appendix F, Appendix G, Appendix H, Appendix I.

## 2. On the Applications of Our Method and the Current State of the Art

**Figure 1:**
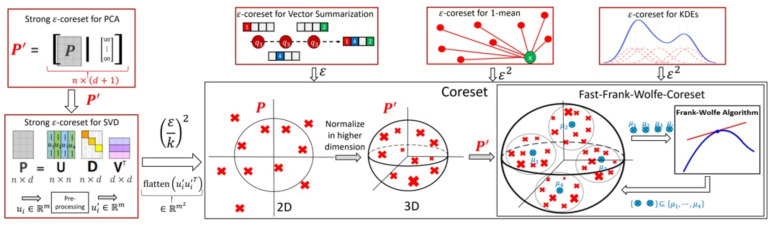
Illustration of Algorithm 2, its normalization of the input, its main applications (red boxes) and their plugged parameters. Algorithm 2 utilizes and boosts the run-time of the Frank–Wolfe algorithm for those applications; see Section 1.4.

In what follows, we will present some of the applications of our theoretical contributions as well as discussing the current state of the art coreset/sketch methods in terms of running time for each of application. Figure 1 summarizes the main applications of our result.

**Figure 2:**
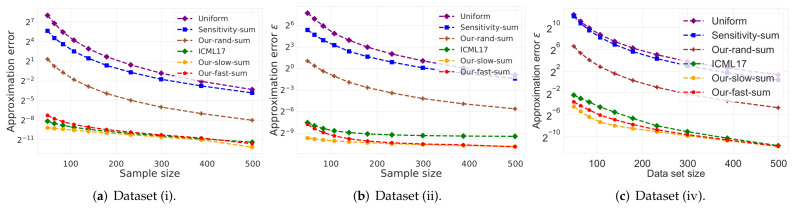
Experimental results for vector summarization. The *x* axis is the size of the subset (coreset), while the *y* axis is the approximation error ${∥\mu -\mu_{s}∥}^{2}$. The difference between the two graphs is the chosen dataset.

**Figure 3:**
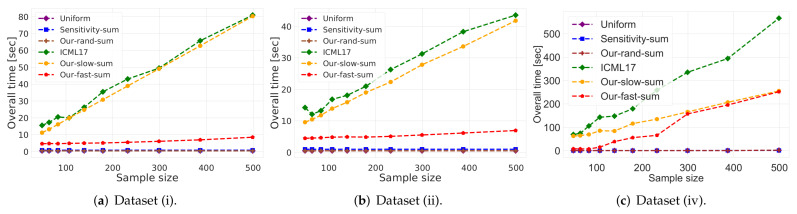
Experimental results for vector summarization. The *x* axis is the size of the subset (coreset), while the *y* axis is the overall time took to compute the coreset and to solve the problem on it. The difference between the two graphs is the chosen dataset.

(i) **Vector summarization:** the goal is to maintain the sum of a (possibly infinite) stream of vectors in $R^{d}$, up to an additive error of ε multiplied by their variance. This is a generalization of frequent items/directions [28].
  As explained in [29], the main real-world application is extractions and compactly representing groups and activity summaries of users from underlying data exchanges. For example, GPS traces in mobile networks can be exploited to identify meetings, and exchanges of information in social networks sheds light on the formation of groups of friends. Our algorithm tackles these application by providing provable solution to the heavy hitters problem in proximity matrices. The heavy hitters problem can be used to extract and represent in a compact way friend groups and activity summaries of users from underlying data exchanges.
  We propose a deterministic algorithm which reduces each subset of *n* vectors into O(1/ε) weighted vectors in $O(nd+d\log{\left(n\right)}^{2}/\varepsilon^{2})$ time, improving upon the nd/ε of [29] (which is the current state of the art in terms of running time), for a sufficiently large *n*; see Corollary 4, and Figures 2 and 3. We also provide a non-deterministic coreset construction in Lemma 5. The merge-and-reduce tree can then be used to support streaming, distributed or dynamic data.
(ii) **Kernel Density Estimates (KDE):** by replacing ε with $\varepsilon^{2}$ for the vector summarization, we obtain fast construction of an ε-coreset for KDE of Euclidean kernels [17]; see more details in Section 4. Kernel density estimate is a technique for estimating a probability density function (continuous distribution) from a finite set of points to better analyse the studied probability distribution than when using a traditional [30,31].
(iii) **1-mean problem:** a coreset for 1-mean which approximates the sum of squared distances over a set of *n* points to any given center (point) in $R^{d}$. This problem arises in facility location problems (e.g., to compute the optimal location for placing an antenna such that all the customers are satisfied). Our deterministic construction computes such a weighted *subset* of size $O(1/\varepsilon^{2})$ in $O(\min\left\{nd/\varepsilon^{2},nd+d\log{\left(n\right)}^{2}/\varepsilon^{4}\right\})$ time. Previous results of [19,32,33,34] suggested coresets for such problem. Unlike our results, these works are either non-deterministic, the coreset is not a subset of the input, or the size of the coreset is linear in *d*.
(iv) **Coreset for LMS solvers and dimensionality reduction:** for example, a deterministic construction for singular value decomposition (SVD) that gets a matrix $A\in R^{n\times d}$ and returns a weighted subset of $k^{2}/\varepsilon^{2}$ rows, such that their weighted distance to any *k*-dimensional non-affine (or affine in the case of PCA) subspace approximates the distance of the original points to this subspace. The SVD and PCA are very common algorithms (see [35]), and can be used for noise reduction, data visualization, cluster analysis, or as an intermediate step to facilitate other analyses. Thus, improving them might be helpful for a wide range of real-world applications. In this paper, we propose a *deterministic* coreset construction that takes $O(nd^{2}+d^{2}k^{4}\log{\left(n\right)}^{2}/\varepsilon^{4})$ time, improving upon the state of the art result of [35] which requires $O(nd^{2}k^{2}/\varepsilon^{2})$ time; see Table 1. Many non-deterministic coresets constructions were suggested for those problems, the construction techniques apply non-uniform sampling [36,37,38], Monte-Carlo sampling [39], and leverage score sampling [23,40,41,42,43,44,45].

## 3. Vector Summarization Coreset

**Notation** **1.** *We denote by $\left[n\right]=\left\{1,\cdots ,n\right\}$. For a vector $v\in R^{d}$, the 0-norm is denoted by ${∥v∥}_{0}$ and is equal to the number of non-zero entries in v. We denote by $e_{\left(i\right)}$ the ith standard basis vector in $R^{n}$ and by 0 the vector ${\left(0,\cdots ,0\right)}^{T}\in R^{n}$. A vector $w\in {\left[0,1\right]}^{n}$ is called a distribution vector if all its entries are non-negative and sums up to one. For a matrix $A\in R^{m\times n}$ and i∈[m],j∈[n] we denote by $A_{i,j}$ the jth entry of the ith row of A. A weighted set is a pair (Q,m) where $Q=\left\{q_{1},\cdots ,q_{n}\right\}\subseteq R^{d}$ is a set of n*points*, and $m={\left(m_{1},\cdots ,m_{n}\right)}^{T}\in R^{n}$ is a weights vector that assigns every $q_{i}\in Q$ a weight $m_{i}\in R$. A matrix $A\in R^{d^{\prime }\times d}$ is orthogonal if $A^{T}A=I\in R^{d\times d}$.*

**Adaptations.** To adapt to the notation of the following sections and the query space (P,w,X,f) to the techniques that we use, we restate (4) as follows. Previously, we denote the queries $X=\left\{x_{1},\cdots ,x_{d}\right\}$, and the input set by $P=\left\{p_{1},\cdots ,p_{n}\right\}$. Now, each input point $p_{i}$ in the input set *P* corresponds to a point $q_{i}=\left(f\left(p_{i},x_{1}\right),\cdots ,f\left(p_{i},x_{d}\right)\right)\in R^{d}$, i.e., each entry of $q_{i}$ equals to $f(p_{i},x)$ for a different query *x*. Throughout the rest of the paper, for technical reason and simplicity, we might alternate between the weights function notation and a weights vector notation. In such cases, the weights function w:P→[0,∞) and weight $w(q_{i})$ of $q_{i}$, i∈[m] are replaced by a vector of weights $m=\left(m_{1},\cdots ,m_{n}\right)\in {\left[0,\infty \right)}^{n}$ and $m_{i}$, respectively, and vice versa. In such cases, the ε-sample is represented by a sparse vector u∈[0,∞) where $S=\left\{p_{i}\in P|u_{i}>0,i\in \left[n\right]\right\}$ is the chosen subset of *P*.

Hence, $f_{w}\left(P,X\right)=\sum_{p\in P}w\left(p\right)\left(f\left(p,x_{1}\right),\cdots ,f\left(p,x_{d}\right)\right)=\sum_{i=1}^{n}m_{i}q_{i}$, and $f_{u}\left(S,X\right)=\sum_{i=1}^{n}u_{i}q_{i}$.

**From $\left(\varepsilon ,{∥\cdot ∥}_{2}\right)$-samples to ε-coresets.** We now define an ε-coreset for vector summarization, which is a re-weighting of the input weighted set (Q,m) by a new weights vector *u*, such that the squared norm of the difference between the weighted means of (Q,u) and (Q,m) is small. This relates to Section 1.3, where an $\left(\sqrt{\varepsilon },{∥\cdot ∥}_{2}\right)$-sample there (in Section 1.3) is an ε-coreset for the vector summarization here.

**Definition** **1** (vector summarization ε-coreset). *Let (Q,m) and (Q,u) be two weighted sets of n points in $R^{d}$, and let ε∈[0,1). Let $\mu =\sum_{i=1}^{n}\frac{m_{i}}{{∥m∥}_{1}}q_{i}$, $\sigma^{2}=\sum_{i=1}^{n}\frac{m_{i}}{{∥m∥}_{1}}{∥q_{i}-\mu ∥}^{2}$, and $\tilde{\mu }=\sum_{i=1}^{n}\frac{u_{i}}{{∥u∥}_{1}}q_{i}$. Then (Q,u) is a*vector summarization ε-coreset*for (Q,m) if ${∥\tilde{\mu }-\mu ∥}_{2}^{2}\leq \varepsilon \sigma^{2}$.*

**Analysis flow.** In what follows we (first) assume that the points of our input set *P* lie inside the unit ball ($\forall_{p\in P}:∥p∥\leq 1$). For such an input set, we present a construction of a variant of a vector summarization coreset, where the error is ε and does not depend on the variance of the input. This construction is based on the Frank–Wolfe algorithm [48]; see Theorem 1 and Algorithm 1. This is by reducing the problem to the problem of maximizing a concave function f(x) over every vector in the unit simplex. Such problems can be solved approximately by a simple greedy algorithm known as the Frank–Wolfe algorithm.
**Algorithm 1:**FRANK–WOLFE(f,K); Algorithm 1.1 of [48]1:**Input:** A concave function $f:R^{n}\to R$, and the number of iterations *K*.2:**Output:** A vector $x\in R^{n}$ that satisfies Theorem 1.3:$x_{\left(0\right)}:=$ the unit *n*-simplex vertex with largest *f* value.4:**for**$k\in \left\{0,\cdots ,K\right\}$**do**5:$i^{\prime }:={arg max}_{i}\nabla f{\left(x_{\left(k\right)}\right)}_{i}$6:$\alpha^{\prime }:=\underset{\alpha_{\left[0,1\right]}}{arg max}f\left(x_{\left(k\right)}+\alpha \left(e_{\left(i^{\prime }\right)}-x_{\left(k\right)}\right)\right)$7:$x_{\left(k+1\right)}:=x_{\left(k\right)}+\alpha^{\prime }\left(e_{\left(i^{\prime }\right)}-x_{\left(k\right)}\right)$8:**end for**9:**Return $x_{\left(k+1\right)}$**

We then present a proper coreset construction in Algorithm 2 and Theorem 2 for a general input set *Q* in $R^{d}$. This algorithm is based on a reduction to the simpler case of points inside the unit ball; see Figure 1 for illustration. This reduction is inspired by the sup-sampling (see Section 1), there (in Section 1) the functions are normalized (to obtain values in [−1,1]) and reweighted (to obtain a non-biased estimator), then the bounds were easily obtained using the Hoeffding inequality. Here, we apply different normalizations and reweightings, and instead of the non-deterministic Hoeffding inequality, we suggest a deterministic version using the Frank–Wolfe algorithm. Our new suggested normalizations (and reweightings) allow us to generalize the result to many more applications as in Section 4.

For brevity purposes, all proofs of the technical results can be found at the Appendix A, Appendix B, Appendix C, Appendix D, Appendix E, Appendix F, Appendix G, Appendix H, Appendix I.

**Theorem** **1** (Coreset for points in the unit ball)**.** *Let $P=\{p_{1},\cdots ,p_{n}\}$ be a set of n points in $R^{d}$ such that $∥p_{i}∥\leq 1$ for every i∈[n]. Let ε∈(0,1) and $w={\left(w_{1},\cdots ,w_{n}\right)}^{T}\in {\left[0,1\right]}^{n}$ be a distribution vector. For every $x={\left(x_{1},\cdots ,x_{n}\right)}^{T}\in R^{n}$, define $f\left(x\right)=-{∥\sum_{i=1}^{n}\left(w_{i}-x_{i}\right)p_{i}∥}^{2}$. Let $\tilde{u}$ be the output of a call to $Frank-Wolfe(f,\left\lceil \frac{8}{\varepsilon }\right\rceil )$; see Algorithm 1. Then:*

*(i)*  *$\tilde{u}$ is a distribution vector with ${∥\tilde{u}∥}_{0}\leq \left\lceil \frac{8}{\varepsilon }\right\rceil$,*
*(ii)*  *${∥\sum_{i=1}^{n}\left(w_{i}-\tilde{u_{i}}\right)p_{i}∥}^{2}\leq \varepsilon$, and*
*(iii)*  *$\tilde{u_{i}}$ is computed in $O\left(\frac{nd}{\varepsilon }\right)$ time.*

We now show how to obtain a vector summarization ε-coreset of size O(1/ε) in $O(\frac{nd}{\varepsilon })$ time for any set $P\subseteq R^{d}$.

**Theorem** **2** (Vector summarization coreset)**.** *Let (Q,m) be a weighted set of n points in $R^{d}$, ε∈(0,1), and let u be the output of a call to $CoreSet(Q,m,\frac{\varepsilon }{16})$; see Algorithm 2. Then, $u\in R^{n}$ is a vector with ${∥u∥}_{0}\leq \frac{128}{\varepsilon }$ non-zero entries that is computed in $O(\frac{nd}{\varepsilon })$ time, and (Q,u) is a vector summarization ε-coreset for (Q,m).*

**Algorithm 2:**
CORESET

(Q,m,ε)


1:**Input:** A weigthed set (Q,m) of n≥2 points in $R^{d}$ and an error parameter ε∈(0,1).2:**Output:** A weight vector $u\in {\left[0,\infty \right)}^{n}$ with O(1/ε) non-zero entries that satisfies Theorem 2.3:

$w:=\frac{m}{{∥m∥}_{1}}$

4:

$\mu_{w}:=\sum_{i=1}^{n}w_{i}q_{i}$

5:

$\sigma_{w}:=\sqrt{\sum_{i=1}^{n}w_{i}{∥q_{i}-\mu ∥}^{2}}$

6:**for** every i∈{1,⋯,n} **do**7:

$p_{i}:=\frac{q_{i}-\mu }{\sigma }$

8:$p_{i}^{\prime }:=\frac{{\left(p_{i}^{T}|1\right)}^{T}}{{∥(p_{i}^{T}|1)∥}^{2}}$       {Notice: $∥p_{i}^{\prime }∥\leq 1$.}9:

$w_{i}^{\prime }:=\frac{w_{i}{∥(p_{i}^{T}|1)∥}^{2}}{2}$

10:
**end for**
11:Compute a sparse vector $u^{\prime }$ with O(1/ε) non-zero entries, such that ${∥\sum_{i=1}^{n}\left(w_{i}^{\prime }-u_{i}^{\prime }\right)p_{i}^{\prime }∥}^{2}\leq \varepsilon$ {E.g., using Algorithm 1 (see Theorem 1).}12:$u_{i}:={∥m∥}_{1}\cdot \frac{2u_{i}^{\prime }}{{∥(p_{i}^{T}|1)∥}^{2}}$    for every $i\in \left\{1,\cdots ,n\right\}$13:
**Return** *u*



### 3.1. Boosting the Coreset’s Construction Running Time

In this section, we present Algorithm 3, which aims to boost the running time of Algorithm 1 from the previous section; see Theorem 3. The main idea behind this new boosted algorithm is as follows: instead of running the Frank–Wolfe algorithm on a (full) set of input data, it can be more efficient to partition the input into a constant number *k* of equal-sized chunks, pick some representative for each chunk (its mean), run the Frank–Wolfe algorithm only on the set of representatives (the set of means) to obtain back a subset of those representative, and then continue recursively only with the chunks whose representative was chosen by the algorithm. Although the Frank–Wolfe algorithm is now applied multiple times (rather than once), each of those runs is much more efficient since only the small set of representatives is considered.

This immediately implies a faster construction time of vector summarization ε-coresets for general input sets; see Corollary 4 and Figure 1 for illustration.

**Theorem** **3** (Faster coreset for points in the unit ball)**.** *Let P be a set of n points in $R^{d}$ such that $∥p∥\leq 1$ for every p∈P. Let w:P→(0,1) be a weights function such that $\sum_{p\in P}w\left(p\right)=1$, ε∈(0,1), and let (C,u) be the output of a call to Fast−FW−CoreSet(P,w,ε); see Algorithm 3. Then*

*(i)*  *|C|≤8/ε and $\sum_{p\in C}u\left(p\right)=1$,*
*(ii)*  *${∥\sum_{p\in P}w\left(p\right)p-\sum_{p\in C}u\left(p\right)p∥}^{2}\leq 2\varepsilon$, and*
*(iii)*  *(C,u) is computed in $O\left(nd+\frac{d\cdot \log{\left(n\right)}^{2}}{\varepsilon^{2}}\right)$ time.*

**Corollary** **4** (Faster vector summarization coreset). *Let (Q,m) be a weighted set of n points in $R^{d}$, and let ε∈(0,1). Then in $O\left(nd+d\cdot \frac{\log{\left(n\right)}^{2}}{\varepsilon^{2}}\right)$ time, we can compute a vector $u={\left(u_{1},\cdots ,u_{n}\right)}^{T}\in R^{n}$, such that u has ${∥u∥}_{0}\leq 128/\varepsilon$ non-zero entries and (Q,u) is a vector summarization (2ε)-coreset for (Q,m).*

**Algorithm 3:**
FAST-FW-CORESET

(P,w,ε)


1:**Input:** A weighed set (P,w) of n≥2 points in $R^{d}$ and an error parameter ε∈(0,1).2:**Output:** A pair (C,u) that satisfies Theorem 33:

$k:=\frac{2\log(n)}{\varepsilon }$

4:
**if** |P|≤k **then**
5:**Return:** A vector summarization ε-corset for (P,w) using Theorem 1.6:
**end if**
7:$\left\{P_{1},\cdots ,P_{k}\right\}:=$ a partition of *P* into *k* disjoint subsets, each contains at most $\left\lceil n/k\right\rceil$ points.8:**for** every $i\in \left\{1,\cdots ,k\right\}$ **do**9:$\mu_{i}:=\frac{1}{\sum_{q\in P_{i}}w\left(q\right)}\cdot \sum_{p\in P_{i}}w\left(p\right)\cdot p$ {The weighted mean of $P_{i}$}10:

$w^{\prime }\left(\mu_{i}\right):=\sum_{p\in P_{i}}w\left(p\right)$

11:
**end for**
12:$(\tilde{\mu },\tilde{u}):=$ a vector summarization $\left(\frac{\varepsilon }{\log(n)}\right)$-corset for the weighted set $\left(\left\{\mu_{1},\cdots ,\mu_{k}\right\},w^{\prime }\right)$ using Theorem 1.13:$C:=\underset{\mu_{i}\in \tilde{\mu }}{⋃}P_{i}$ {*C* is the union over all subsets $P_{i}$ whose mean $\mu_{i}$ was chosen in $\tilde{\mu }$.}14:**for** every $\mu_{i}\in \tilde{\mu }$ and $p\in P_{i}$ **do**15:

$u\left(p\right):=\frac{\tilde{u}\left(\mu_{i}\right)w\left(p\right)}{\sum_{q\in P_{i}}w\left(q\right)}$

16:
**end for**
17:

(C,u):=Fast−FW−CoreSet(C,u,ε)

18:
**Return:** (C,u)




In what follows, we show how to compute a vector summarization coreset with high probability in a time that is sublinear in the input size |Q|=n. This is based on the geometric median trick, that suggests the following procedure: (i) sample k>1 sets $\left\{S_{1},\cdots ,S_{k}\right\}$ of the same (small) size from the original input set *Q*, (ii) for each such sampled set $S_{i}$ (i∈[k]), compute its mean ${\bar{s}}_{i}$, and finally, (iii) compute and return the geometric median of those means $\bar{s}=\left\{{\bar{s}}_{1},\cdots ,{\bar{s}}_{k}\right\}$. This geometric median is guaranteed to approximate the mean of the original input set *Q*.

We show that there is no need to compute this geometric median, as it is a difficult computational task. We prove that there exists a set $S_{i^{*}}$ from the sampled subsets such that its mean ${\bar{s}}_{i^{*}}$ is very close to this geometric median, with high probability. Thus, ${\bar{s}}_{i^{*}}$ is a good approximation to the desired mean of the original input set. Furthermore, we show that ${\bar{s}}_{i^{*}}$ is simply the point in $\bar{s}$ that minimizes its sum of (non-squared) distances to this set $\bar{s}$, i.e., $i^{*}\in {arg min}_{j\in [k]}\sum_{i=1}^{k}{∥{\bar{s}}_{i}-{\bar{s}}_{j}∥}_{2}$. An exhaustive search over the points of $\bar{s}$ can thus recover ${\bar{s}}_{i^{*}}$. The corresponding set $S_{i^{*}}$ is the resulted vector summarization coreset; see Lemma 5 and Algorithm 4.

**Lemma** **5** (Fast probabilistic vector summarization coreset). *Let Q be a set of n points in $R^{d}$, $\mu =\frac{1}{n}\sum_{p\in P}q$, and $\sigma^{2}=\frac{1}{n}\sum_{p\in P}{∥q-\mu ∥}^{2}$. Let ε∈(0,1), δ∈(0,0.9], and let $S\subseteq R^{d}$ be the output of a call to Prob−Weak−Coreset(Q,ε,δ); see Algorithm 4. Then:*

*(i)*  *S⊆Q and $\left|S\right|=\frac{4}{\varepsilon }$,*
*(ii)*  *with probability at least 1−3δ we have ${∥\frac{1}{\left|S\right|}\sum_{p\in S}p-\mu ∥}^{2}\leq 33\cdot \varepsilon \sigma^{2}$, and*
*(iii)*  *S is computed in $O\left(d\log{\left(\frac{1}{\delta }\right)}^{2}+\frac{d\log\left(\frac{1}{\delta }\right)}{\varepsilon }\right)$ time.*

**Algorithm 4:**
PROB-WEAK-CORESET

(Q,ε,δ)


1:**Input:** A set *Q* of n≥2 points in $R^{d}$, ε∈(0,1), and δ∈(0,1).2:**Output:** A subset S⊆P that satisfies Lemma 5.3:$k:=3.5\log\left(\frac{1}{\delta }\right)+1$.4:S:= an i.i.d sample of size $\frac{4k}{\varepsilon }$.5:$\left\{S_{1},\cdots ,S_{k}\right\}:=$ a partition of *S* into *k* disjoint subsets, each contains $\frac{4}{\varepsilon }$ points.6:${\bar{s}}_{i}:=$ the mean of the *i*’th subset $S_{i}$ for i∈[k].7:$i^{*}:={arg min}_{j\in [k]}\sum_{i=1}^{k}{∥{\bar{s}}_{i}-{\bar{s}}_{j}∥}_{2}$.8:
**Return** $S_{i^{*}}$




## 4. Applications

**Coreset for 1-mean.** A 1-mean ε-coreset for (Q,m) is a weighted set (Q,u) such that for every $x\in R^{d}$, the sum of squared distances from *x* to either (Q,m) or (Q,u), is approximately the same. To maintain the above property, we prove that it suffices for (Q,u) to satisfy the following: the mean, the variance, and the sum of weights of (Q,u) should approximate the mean, the variance, and the sum of weights of (Q,m), respectively, up to an additive error that depends linearly on ε. Then note that when plugging $\varepsilon^{2}$ (rather than ε) as input to Algorithm 2, the output is guaranteed to satisfy the above 3 properties, by construction of *u*.

The following theorem computes a 1-mean ε-coreset.

**Theorem** **6.** *Let (Q,m) be a weighted set of n points in $R^{d}$, ε∈(0,1). Then in*

$$O\left(\min\left\{nd+d\cdot \frac{\log{\left(n\right)}^{2}}{\varepsilon^{4}},\frac{nd}{\varepsilon^{2}}\right\}\right)$$

*time we can compute a vector $u={\left(u_{1},\cdots ,u_{n}\right)}^{T}\in R^{n}$, where ${∥u∥}_{0}\leq \frac{128}{\varepsilon^{2}}$, such that:*

$$\forall x\in R^{d}:\;\left|\sum_{i=1}^{n}\left(m_{i}-u_{i}\right){∥q_{i}-x∥}^{2}\right|\leq \varepsilon \sum_{i=1}^{n}m_{i}{∥q_{i}-x∥}^{2}.$$

**Coreset for KDE.** Given two sets of points *Q* and $Q^{\prime }$, and a kernel $K:R^{d}\times R^{d}\to R$ that is defined by the kernel map ϕ, the maximal difference

$$\underset{x\in R^{d}}{\sup}\left|\sum_{q\in Q}\frac{K(x,q)}{\left|Q\right|}-\sum_{q^{\prime }\in Q^{\prime }}\frac{K(x,q^{\prime })}{|Q^{\prime }|}\right|$$

between the kernel costs of *Q* and $Q^{\prime }$ is upper bounded by ${∥\mu_{\hat{Q}}-\mu_{\hat{Q^{\prime }}}∥}_{2}$, where $\mu_{\hat{Q}}$ and $\mu_{\hat{Q^{\prime }}}$ are the means of $\hat{Q}=\left\{\phi (q)|q\in Q\right\}$ and ${\hat{Q}}^{\prime }=\left\{\phi \left(q\right)|q\in Q^{\prime }\right\}$, respectively, [49]. Given $\hat{Q}$, we can compute a vector summarization $\varepsilon^{2}$-coreset ${\hat{Q}}^{\prime }$, which satisfies that ${∥\mu_{\hat{Q}}-\mu_{\hat{Q^{\prime }}}∥}_{2}^{2}\leq \varepsilon^{2}$. By the above argument, this is also an ε-KDE coreset.

**Coreset for dimensionality reduction and LMS solvers.** An ε-coreset for the *k*-SVD (*k*-PCA) problem of *Q* is a small weighted subset of *Q* that approximates the sum of squared distances from the points in *Q* to every non-affine (affine) *k*-dimensional subspace of $R^{d}$, up to a multiplicative factor of 1±ε; see Corollary 7. Coreset for LMS solvers is the special case of k=d−1.

In [35], it is shown how to leverage an ${\left(\varepsilon /k\right)}^{2}$-coreset for the vector summarization problem in order to compute an ε-coreset for *k*-SVD. In [45], it is shown how to compute a coreset for *k*-PCA via a coreset for *k*-SVD, by simply adding another entry with some value r∈R to each vector of the input. Algorithm 5 combines both the above reductions, along with a computation of a vector summarization ${\left(\varepsilon /k\right)}^{2}$-coreset to compute the desired coreset for dimensionality reduction (both *k*-SVD and *k*-PCA). To compute the vector summarization coreset we utilize our new algorithms from the previous sections, which are faster than the state of the art algorithms.
**Algorithm 5:**DIM-CORESET(A,k,ε)1:**Input:** A matrix $A\in R^{n\times d}$, an integer k∈[d], and an error parameter ε∈(0,1).2:**Output:** A diagonal matrix $W\in R^{n\times n}$ that satisfies Corollary 7.3:$r:=1+\max_{i\in [n]}\frac{4{∥a_{i}∥}^{2}}{\varepsilon^{4}}$ {where $a_{i}$ is the *i*th row of *A*}4:U,Σ,V: = the full SVD of $\left[A|{\left(r,\cdots ,r\right)}^{T}\right]\in R^{n\times (d+1)}$5:$v_{i}:=\left(U_{i,1},\cdots ,U_{i,k},\frac{U_{i,k+1:d}\Sigma_{k+1:d,k+1:d}}{{∥\Sigma_{k+1:d,k+1:d}∥}_{F}}\right)$ for every i∈[n]6:$\tilde{v_{i}}$:= the row stacking of $v_{i}v_{i}^{T}\in R^{d\times d}$ for every i∈[n]7:$\left(\left\{\tilde{v_{1}},\cdots ,\tilde{v_{n}}\right\},u\right)$: = a vector summarization ${\left(\frac{\varepsilon }{5k}\right)}^{2}$-coreset for $\left(\left\{\tilde{v_{1}},\cdots ,\tilde{v_{n}}\right\},\left(1,\cdots ,1\right)\right)$.8:W:= a diagonal matrix in $R^{n\times n}$, where $W_{i,i}=\sqrt{u_{i}}$, ∀i∈[n].9:**Return***W*

**Corollary** **7** (Coreset for dimensionality reduction)**.** *Let Q be a set of n points in $R^{d}$, and let $A\in R^{n\times d}$ be a corresponding matrix containing the points of Q in its rows. Let $\varepsilon \in (0,\frac{1}{2})$ be an error parameter, k∈[d] be an integer, and W be the output of a call to DIM−CoreSet(A,k,ε). Then:*

*(i)*  *W is a diagonal matrix with $O\left(\frac{k^{2}}{\varepsilon^{2}}\right)$ non-zero entries,*
*(ii)*  *W is computed in $O\left(\min\left\{nd^{2}+\frac{d^{2}\log{\left(n\right)}^{2}k^{4}}{\varepsilon^{4}},\frac{nd^{2}k^{2}}{\varepsilon^{2}}\right\}\right)$ time, and*
*(iii)*  *there is a constant c, such that for every $\ell \in R^{d}$ and an orthogonal $X\in R^{d\times (d-k)}$ we have*
  $$|1-\frac{{∥W(A-\ell )X∥}_{F}^{2}}{{∥(A-\ell )X∥}_{F}^{2}}|\leq c\varepsilon .$$
  *Here, A−ℓ is the subtraction of ℓ from every row of A.*

**Where do our methods fit in?** Theoretically speaking, the 1-mean problem (also known as the arithmetic mean problem), is a widely used tool for reporting central tendencies in the field of statistics, as it is also used in machine learning. As for the practical aspect of such problem, it can be either used to obtain an estimation of the mathematical expectation of signal strength in a area [50], or as an imputation technique used to fill in missing values, e.g., in the context of filling in missing values of heart monitor sensor data [51]. Note that a variant of this problem is widely used in the context of deep learning, namely, the moving averages. Algorithms 3 and 4 can boost such methods when given large-scale datasets. In addition, our algorithms extend also to SVD, PCA, and LMS where these methods are known for their usages and efficiencies in discovering a low dimensional representation of high dimensional data. From a practical point of view, SVD showed promising results when dealing with on calibration of a star sensor on-orbit calibration [52], denoising a 4-dimensional computed tomography of the brain in stroke patients [53], removal of cardiac interference from trunk electromyogram [54], among many other applications.

We propose a summarization technique (see Algorithm 5) that aims to compute an approximation towards the SVD factorization of large-scale datasets where applying the SVD factorization on the dataset is not possible due to insufficient memory or long computational time.

## 5. Experimental Results

We now apply different coreset construction algorithms presented in this paper to a variety of applications, in order to boost their running time, or reduce their memory storage. We note that a complete open source code is provided [55].

**Software/Hardware.** The algorithms were implemented in Python 3.6 [56] using “Numpy” [57]. Tests were conducted on a PC with Intel i9-7960X CPU @2.80 GHz x 32 and 128 Gb RAM.

**We compare the following algorithms:** (To simply distinguish between our algorithms and the competing ones in the graphs, observe that the labels of our algorithms starts with the prefix “Our-”, while the competing methods do not.)

(i) Uniform: Uniform random sample of the input *Q*, which requires sublinear time to compute.
(ii) Sensitivity-sum: Random sampling based on the “sensitivity” for the vector summarization problem [58]. Sensitivity sampling is a widely known technique [19], which guarantees that a subsample of sufficient size approximates the input well. The sensitivity of a point q∈Q is $\frac{1}{n}+\frac{{∥q∥}^{2}}{\sum_{q^{\prime }\in Q}{∥q^{\prime }∥}^{2}}$. This algorithm takes O(nd) time.
(ii) ICML17: The vector summarization coreset construction algorithm from [29] (see Algorithm 2 there), which runs in O(nd/ε) time.
(iv) Our-rand-sum: Our coreset construction from Lemma 5, which requires $O\left(d\log{\left(\frac{1}{\delta }\right)}^{2}+\frac{d\log\left(\frac{1}{\delta }\right)}{\varepsilon }\right)$ time.
(v) Our-slow-sum: Our coreset construction from Corollary 2, which requires O(nd/ε) time.
(vi) Our-fast-sum: Our coreset construction from Corollary 4, which requires $O(nd+d\log{\left(n\right)}^{2}/\varepsilon^{2})$ time.
(vii) Sensitivity-svd: Similar to Sensitivity-sum above, however, now the sensitivity is computed by projecting the rows of the input matrix *A* on the optimal *k*-subspace (or an approximation of it) that minimizes its sum of squared distances to the rows of *A*, and then computing the sensitivity of each row *i* in the projected matrix $A^{\prime }$ as ${∥u_{i}∥}^{2}$, where $u_{i}$ is the *i*th row the matrix *U* from the SVD of $A^{\prime }=UDV^{T}$; see [37]. This takes O(ndk) time.
(viii) NIPS16: The coreset construction algorithm from [35] (see Algorithm 2 there) which requires $O(nd^{2}k^{2}/\varepsilon^{2})$ time.
(ix) Our-slow-svd: Corollary 7 offers a coreset construction for SVD using Algorithm 5, which utilizes Algorithm 2. However, Algorithm 2 either utilizes Algorithm 1 (see Theorem 2) or Algorithm 3 (see Theorem 4). Our-slow-svd applies the former option, which requires $O(nd^{2}k^{2}/\varepsilon^{2}))$ time.
(x) Our-fast-svd: Corollary 7 offers a coreset construction for SVD using Algorithm 5, which utilizes Algorithm 2. However, Algorithm 2 either utilizes Algorithm 1 (see Theorem 2) or Algorithm 3 (see Theorem 4). Our-fast-svd uses the latter option, which requires $O(nd^{2}+d^{2}\log{\left(n\right)}^{2}k^{4}/\varepsilon^{4})$ time.

**Datasets.** We used the following datasets from the UCI ML library [59]:

(i) New York City Taxi Data [60]. The data covers the taxi operations at New York City. We used the data describing n=14.7M trip fares at the year of 2013. We used the d=6 numerical features (real numbers).
(ii) US Census Data (1990) [61]. The dataset contains n=2.4M entries. We used the entire d=68 real-valued attributes of the dataset.
(iii) Buzz in social media Data Set [62]. It contains n=0.5M examples of buzz events from two different social networks: Twitter, and Tom’s Hardware. We used the entire d=77 real-valued attributes.
(iv) Gas Sensors for Home Activity Monitoring Data Set [63]. This dataset has n=919,438 recordings of a gas sensor array composed of 8 MOX gas sensors, and a temperature and humidity sensor. We used the last d=10 real-valued attributes of the dataset.

**Discussion regarding the chosen datasets.** The Buzz in social media data set is widely used in the context of Principal Component Regression (or, PCR in short), that is used for estimating the unknown regression coefficients in a standard linear regression model. The goal of PCR in the context of this dataset, is to predict popularity of a certain topic on Twitter over a period. It is known that the solution of the PCR problem can be approximated using the known SVD decomposition problem. Our techniques enable us to benefit from the coreset advantages, e.g., to boost the PCR approximated solution (PCA) while using low memory, and supporting the streaming model by maintaining a coreset for the data (tweets) seen so far; each time a new point (tweet) is received, it is added to current stored coreset in memory. Once the stored coreset is large enough, our compression (coreset construction algorithm) is applied. This procedure is repeated until the stream of points is empty.

The New York City taxi data contains information about the locations of passengers as well as the locations of their destinations. Thus, the goal is to find a location which is close to the most wanted destinations. This problem can be formulated as a facility location problem, which can be reduced to an instance of the 1-mean problem. Hence, since our methods admit faster solution as well as provable approximation for the facility location problem, we can leverage our coreset to speed up the computations using this dataset.

Finally, regarding the remaining datasets, PCA has been widely used either for low-dimensional embedding or, e.g., to compute the arithmetic mean. By using our methods, we can boost the PCA while admitting an approximated solution.

**The experiments.**

**Figure 4:**
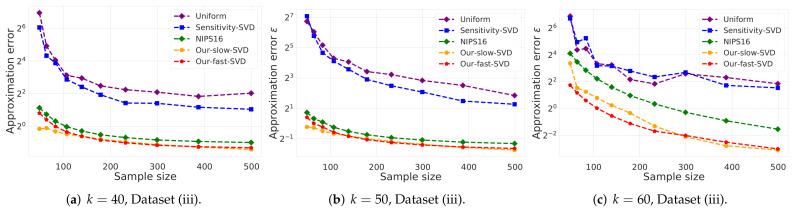
Experimental results for *k*-SVD, we used Dataset (iii). The *x* axis is the size of the subset (coreset), while the *y* axis is the approximation error ε. The difference between the 3 graphs is the chosen low dimension *k*.

**Figure 5:**
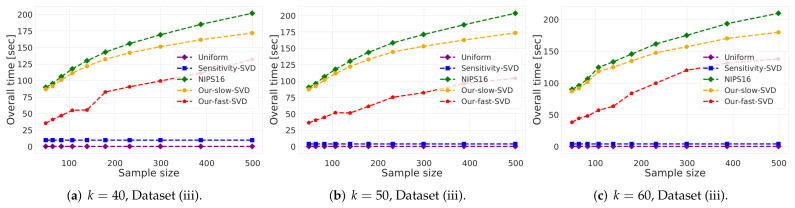
Experimental results for *k*-SVD, we used Dataset (iii). The *x* axis is the size of the subset (coreset), while the *y* axis is the overall time took to compute the coreset and to solve the problem on it. The difference between the 3 graphs is the chosen low dimension *k*.

**Figure 6:**
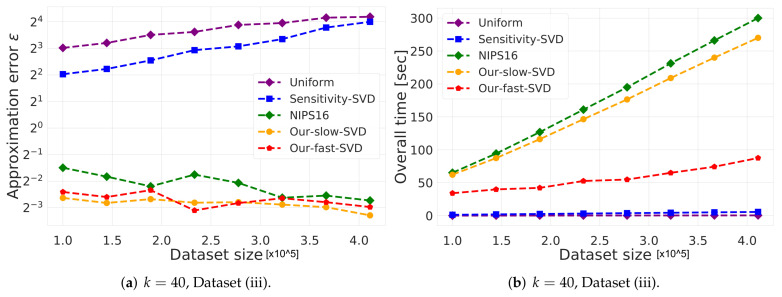
Experimental results for *k*-SVD, we used Dataset (iii). The *x* axis is the size of the dataset which we compress to subsample of size 150, while the *y*-axis is the approximation error in the left hand side graph, and in the right hand side it is the overall time took to compute the coreset and to solve the problem on it.

(i) **Vector summarization:**The goal is to approximate the mean of a huge input set, using only a small weighted subset of the input. The empirical approximation error is defined as ${∥\mu -\mu_{s}∥}^{2}$, where μ is the mean of the full data and $\mu_{s}$ is the mean of the weighted subset computed via each compared algorithm; see Figure 2 and Figure 3.
  In Figure 2, we report the empirical approximation error ${∥\mu -\mu_{s}∥}^{2}$ as a function of the subset (coreset) size, for each of the datasets (i)–(ii), while in Figure 3 we report the overall computational time for computing the subset (coreset) and for solving the 1-mean problem on the coreset, as a function of the subset size.
(ii) ***k*-SVD:**The goal is to compute the optimal *k*-dimensional non-affine subspaces of a given input set. We can either compute the optimal subspace using the original (full) input set, or using a weighted subset (coreset) of the input. We denote by $S^{*}$ and $S^{\prime }$ the optimal subspace when computed either using the full data or using the subset at hand, respectively. The empirical approximation error is defined as the ratio $|\left(c^{*}-c^{\prime }\right)/c^{*}|$, where $c^{*}$ and $c^{\prime }$ are the sum of squared distances between the points of original input set to $S^{*}$ and $S^{\prime }$, respectively; see Figure 4, Figure 5 and Figure 6. Intuitively, this ratio represents the relative SSD error of recovering an optimal *k*-dimensional non-affine subspace on the compression, rather than using the full data.
  In Figure 4 we report the empirical error $|\left(c^{*}-c^{\prime }\right)/c^{*}|$ as a function of the coreset size. In Figure 5 we report the overall computational time in took to compute the coreset and to recover the optimal subspace using the coreset, as a function of the coreset size. In both figures we have three subfigures, each one for a different chosen value of *k* (the dimension of the subspace). Finally, in Figure 6 the *x* axis is the size of the dataset (which we compress to a subset of size 150), while the *y*-axis is the approximation error on the left hand side graph, and on the right hand side it is the overall computational time it took to compute the coreset and to recover the optimal subspace using the coreset.

### 5.1. Discussion

**Vector summarization experiment:**As predicted by the theory and as demonstrated in Figure 2 and Figure 3, our fast and deterministic algorithm Our-fast-sum (the red line in the figures) achieves either the same or smaller approximation errors in most cases compared to the deterministic alternatives Our-slow-sum (orange line) and ICML17 (green line), while being up to ×10 times faster. Hence, when we seek a fast time deterministic solution for computing a coreset for the vector summarization problem, our algorithm Our-fast-sum is the favorable choice.

Compared to the randomized alternatives, Our-fast-sum is obviously slower, but achieves an error more than 3 orders of magnitude smaller. However, our fast and randomized algorithm Our-rand-sum (brown line) constantly achieves better results compared to the other randomized alternatives; It yields approximation error up to ×50 smaller, while maintaining the same computational time. This is demonstrated on both datasets. Hence, our compression can be used to speed up tasks, e.g., computing the PCA or PCR, as described above.

***k*-SVD experiment:** Here, in Figure 4, Figure 5 and Figure 6 we witness a similar phenomena, where our fast and deterministic algorithm Our-fast-svd achieves the same or smaller approximation errors compared to the deterministic alternatives Our-slow-svd and NIPS16, respectively, while being up to ×4 times faster. Compared to the randomized alternatives, Our-fast-svd is slower as predicted, but achieves an error up to 2 orders of magnitude smaller. This is demonstrated for increasing sample sizes (as in Figure 4 and Figure 5), for increasing dataset size (as in Figure 6), and for various values of *k* (see Figure 4, Figure 5 and Figure 6).

### 5.2. Conclusions and Future Work

This paper generalizes the definition of ε-sample and coreset from the worst case error over every query to average $\ell_{2}$ error. We then showed a reduction from the problem of computing such coresets to the vector summarization coreset construction problem. Here, we suggest deterministic and randomized algorithms for computing such coresets, the deterministic version takes $O(\min\left\{nd/\varepsilon ,nd+d\log{\left(n\right)}^{2}/\varepsilon^{2}\right\})$, and the randomized $O(d\log{\left(\frac{1}{\delta }\right)}^{2}+\frac{d\log\left(\frac{1}{\delta }\right)}{\varepsilon })$. Finally, we showed how to leverage an $\left(\varepsilon^{2}\right)$-coreset for the vector summarization problem in order to compute an ε-coreset for the 1-mean problem, and similarly for *k*-SVD and *k*-PCA problem via computing an ${\left(\varepsilon /k\right)}^{2}$ vector summarization coreset after some reprocessing on the data.

Open problems include generalizing these results for other types of norms, or other functions such as M-estimators that are robust to outliers. We hope that the source code and the promising experimental results would encourage also practitioners to use these new types of approximations. Normalization via this new sensitivity type reduced the bounds on the number of iterations of the Frank–Wolfe algorithm by orders of magnitude. We believe that it can be used more generally for provably faster convex optimization, independently of coresets or ε-samples. We leave this for future research. 

## Figures and Tables

**Table 1:** Known deterministic subset coresets for LMS solvers. Our result has the fastest running time for sufficiently large *n* and *d*.

Error	Size	Time	Citation	Notes
ε	$O(k^{2}/\varepsilon^{2})$	$O(nd^{2}k^{2}/\varepsilon^{2})$	[35]	N/A
ε	$O(d/\varepsilon^{2})$	poly(n,d,ε)	[46]	inefficient for large *n*
0	$O(d^{2})$	$O(nd^{2}+\log\left(n\right)poly\left(d\right))$	[22]	inefficient for large *d*
ε	$O(k/\varepsilon^{2})$	poly(n,d,k,ε)	[47]	inefficient for large *n*
ε	$O(k^{2}/\varepsilon^{2})$	$O(nd^{2}+\log{\left(n\right)}^{2}d^{2}k^{4}/\varepsilon^{4})$	🟉	N/A

## Appendix A. Problem Reduction for Vector Summarization ε-Coresets

First, we define a normalized weighted set, which is simply a set which satisfies three properties: weights sum to one, zero mean, and unit variance.

**Definition** **A1** (Normalized weighted set). *A normalized weighted set is a weighted set (P,w) where $P=\left\{p_{1},\cdots ,p_{n}\right\}\subseteq R^{d}$ and $w={\left(w_{1},\cdots ,w_{n}\right)}^{T}\in R^{n}$ satisfy the following properties:*

(a) *Weights sum to one: $\sum_{i=1}^{n}w_{i}=1$,*
(b) *The weighted sum is the origin: $\sum_{i=1}^{n}w_{i}p_{i}=0$, and*
(c) *Unit variance: $\sum_{i=1}^{n}w_{i}{∥p_{i}∥}^{2}=1$.*

### Appendix A.1. Reduction to Normalized Weighted Set

In this section, we argue that in order to compute a vector summarization ε-coreset for an input weighted set (Q,m), it suffices to compute a vector summarization ε-coreset for its corresponding normalized (and much simpler) weighted set (P,w) as in Definition A1; see Corollary A1. However, first, in Observation A1, we show how to compute a corresponding normalized weighted set (P,w) for any input weighted set (Q,m).

**Observation** **A1.**
*Let $Q=\{q_{1},\cdots ,q_{n}\}$ be a set of n≥2 points in $R^{d}$, $m\in {\left(0,\infty \right)}^{n}$, $w\in {\left(0,1\right]}^{n}$ be a distribution vector such that $w=\frac{m}{{∥m∥}_{1}}$, $\mu =\sum_{i=1}^{n}w_{i}q_{i}$ and $\sigma =\sqrt{\sum_{i=1}^{n}w_{i}{∥q_{i}-\mu ∥}^{2}}$. Let $P=\left\{p_{1},\cdots ,p_{n}\right\}$ be a set of n points in $R^{d}$, such that for every j∈[n] we have $p_{j}=\frac{q_{j}-\mu }{\sigma }$. Then, (P,w) is the corresponding normalized weighted set of (Q,m), i.e., (i)–(iii) hold as follows:*

(i) $\sum_{i=1}^{n}w_{i}=1$,
(ii) $\sum_{i=1}^{n}w_{i}p_{i}=0$, and
(iii) $\sum_{i=1}^{n}w_{i}{∥p_{i}∥}^{2}=1$.

**Proof.** 

$$\sum_{i=1}^{n}w_{i}=1,\;\mathrm{immediately}\;\mathrm{holds}\;\mathrm{by}\;\mathrm{the}\;\mathrm{definition}\;\mathrm{of}\;w.$$

$$\begin{array}{cc} \sum_{i=1}^{n}w_{i}p_{i} & =\sum_{i=1}^{n}w_{i}\cdot \frac{q_{i}-\mu }{\sigma }=\frac{1}{\sigma }\left(\sum_{i=1}^{n}w_{i}q_{i}-\sum_{i=1}^{n}w_{i}\mu \right)\\ & =\frac{1}{\sigma }\left(\mu -\sum_{i=1}^{n}w_{i}\mu \right)=\frac{1}{\sigma }\mu \left(1-\sum_{i=1}^{n}w_{i}\right)=0, \end{array}$$

where the first equality holds by the definition of $p_{i}$, the third holds by the definition of μ, and the last is since *w* is a distribution vector.

$$\begin{array}{cc} \sum_{i=1}^{n}w_{i}{∥p_{i}∥}^{2} & =\sum_{i=1}^{n}w_{i}{∥\frac{q_{i}-\mu }{\sigma }∥}^{2}=\frac{1}{\sigma^{2}}\sum_{i=1}^{n}w_{i}{∥q_{i}-\mu ∥}^{2}\\ & =\frac{\sum_{i=1}^{n}w_{i}{∥q_{i}-\mu ∥}^{2}}{\sum_{i=1}^{n}w_{i}{∥q_{i}-\mu ∥}^{2}}=1, \end{array}$$

where the first and third equality hold by the definition of $p_{i}$ and σ, respectively. □

**Corollary** **A1.**
*Let (Q,m) be a weighted set, and let (P,w) be its corresponding normalized weighted set as computed in Observation A1. Let (P,u) be a vector summarization ε-coreset for (P,w) and let $u^{\prime }={∥m∥}_{1}\cdot u$. Then $\left(Q,u^{\prime }\right)$ is a vector summarization ε-coreset for (Q,m).*

**Proof.** Put $x\in R^{d}$ and let $y=\frac{x-\mu }{\sigma }$. Now, for every j∈[n], we have that

(A1) $$\begin{array}{cc} {∥q_{j}-x∥}^{2}\; & ={∥\sigma p_{j}+\mu -\left(\sigma y+\mu \right)∥}^{2}\\ \; & ={∥\sigma p_{j}-\sigma y∥}^{2}\\ \; & =\sigma^{2}\left|\right|p_{j}-y{\left|\right|}^{2}, \end{array}$$

where the first equality is by the definition of *y* and $p_{j}$. Let (P,u) be a vector summarization ε-coreset for (P,w). We prove that $\left(Q,u^{\prime }\right)$ is a vector summarization ε-coreset for (Q,m). We observe the following

(A2) $$\begin{array}{cc} \; & {∥\sum_{i=1}^{n}\frac{m_{i}}{{∥m∥}_{1}}q_{i}-\sum_{i=1}^{n}\frac{u_{i}^{\prime }}{{∥u^{\prime }∥}_{1}}q_{i}∥}^{2}={∥\sum_{i=1}^{n}w_{i}q_{i}-\sum_{i=1}^{n}\frac{u_{i}}{{∥u∥}_{1}}q_{i}∥}^{2}\\ & ={∥\sum_{i=1}^{n}\left(w_{i}-\frac{u_{i}}{{∥u∥}_{1}}\right)\left(p_{i}\sigma +\mu \right)∥}^{2}\\ & ={∥\sum_{i=1}^{n}\left(w_{i}-\frac{u_{i}}{{∥u∥}_{1}}\right)p_{i}\sigma +\mu \sum_{i=1}^{n}\left(w_{i}-\frac{u_{i}}{{∥u∥}_{1}}\right)∥}^{2}\\ & ={∥\sum_{i=1}^{n}\left(w_{i}-\frac{u_{i}}{{∥u∥}_{1}}\right)\left(p_{i}\sigma \right)∥}^{2}\leq \varepsilon \sigma^{2} \end{array}$$

where the first equality holds since $w=\frac{m}{{∥m∥}_{1}}$ and $\frac{u^{\prime }}{{∥u^{\prime }∥}_{1}}=\frac{mu}{m{∥u∥}_{1}}=\frac{u_{i}}{{∥u∥}_{1}}$, the second holds by (A1), and the last inequality holds since (P,u) is a vector summarization ε-coreset for (P,w). □

### Appendix A.2. Vector Summarization Problem Reduction

Given a normalized weighted set (P,w) as in Definition A1, in the following lemma we prove that a weighted set (P,u) is a vector summarization ε-coreset for the normalized weighted set (P,w) if and only if the squared $\ell_{2}$ norm of the weighted mean of (P,u) is smaller than ε.

**Lemma** **A2.**
*Let (P,w) be a normalized weighted set of n points in $R^{d}$, ε∈(0,1), and $u\in R^{n}$ be a weight vector. Let $\bar{p}=\sum_{i=1}^{n}\frac{w_{i}}{{∥w∥}_{1}}p_{i}$, $\bar{s}=\sum_{i=1}^{n}\frac{u_{i}}{{∥u∥}_{1}}p_{i}$, and $\sigma^{2}={∥p_{i}-\bar{p}∥}^{2}$. Then, (P,u) is a vector summarization ε-coreset for (P,w), i.e., ${∥\bar{p}-\bar{s}∥}^{2}\leq \varepsilon \sum_{i=1}^{n}w_{i}\sigma^{2}$ if and only if ${∥\bar{s}∥}^{2}\leq \varepsilon$.*

**Proof.** The proof holds since (P,w) is a normalized wighted set, i.e., $\bar{p}=0$, and $\sigma^{2}=1$. □

## Appendix B. Frank–Wolfe Theorem

Here, for completeness we state the Frank–Wolfe Theorem [48]. This theorem will be used in the proof of Theorem 1 that shows how to compute a variant of vector summarization coreset for points inside the unit ball.

To do so, we consider the measure $C_{f}$ defined in [48]; see equality (9) in Section 2.2. For a simplex *S* and concave function *f*, the quantity $C_{f}$ is defined as

(A3) $$\begin{array}{c} C_{f}:=\sup\frac{1}{\alpha^{2}}\left(f\left(x\right)+{\left(y-x\right)}^{T}\nabla f\left(x\right)-f\left(y\right)\right), \end{array}$$

where the supremum is over every *x* and *z* in *S*, and over every α so that y=x+α(z−x) is also in *S*. The set of such α includes [0,1], but α can also be negative.

**Theorem** **A3** 
**(Theorem 2.2 from [** 48 **]).** *For simplex S and concave function f, Algorithm 1 (Algorithm 1.1 from [48]) finds a point $x_{\left(k\right)}$ on a k-dimensional face of S such that*

$$\frac{f\left(x*\right)-f\left(x_{\left(k\right)}\right)}{4C_{f}}\leq \frac{1}{k+3},$$

*for k > 0, where f(x∗) is the optimal value of f.*

## Appendix C. Proof of Theorem 1

**Proof of Theorem** **1.** Let $C_{f}$ be defined for *f* and *S* as in (A3), and let $f(x^{*})$ be the maximum value of *f* in *S*. Based on Theorem A3 we have:

1. $\tilde{u}$ is a point on a $\left\lceil \frac{8}{\varepsilon }\right\rceil$-dimensional face of *S*, i.e., ${∥\tilde{u}∥}_{0}\leq \left\lceil \frac{8}{\varepsilon }\right\rceil$, $u\in S\subset {\left[0,1\right]}^{n}$ and $\sum_{i=1}^{n}\tilde{u_{i}}=1$. Hence, claim (i) of this theorem is satisfied.
2. $\frac{f\left(x^{*}\right)-f\left(x_{\left(k\right)}\right)}{4C_{f}}\leq \frac{1}{k+3},$ for every $k\in \left\{0,\cdots ,\left\lceil \frac{8}{\varepsilon }\right\rceil \right\}.$

Since f(x)≤0 for every x∈S, we have that,

$$f\left(x^{*}\right)=f\left(w\right)=-{∥\sum_{i=1}^{n}\left(w_{i}-w_{i}\right)p_{i}∥}^{2}=0.$$

Define *A* to be the matrix of d×n such that the *i*-th column of *A* is the *i*-th point in *P*, and let $\mu =\sum_{i=1}^{n}w_{i}p_{i}$. We get that

(A4) $$\begin{array}{cc} f(x) & =-{∥\sum_{i=1}^{n}\left(w_{i}-x_{i}\right)p_{i}∥}^{2}=-{∥\mu -\sum_{i=1}^{n}x_{i}p_{i}∥}^{2}\\ & =-{∥\mu ∥}^{2}+2\mu^{T}\left(\sum_{i=1}^{n}x_{i}p_{i}\right)-{∥\sum_{i=1}^{n}x_{i}p_{i}∥}^{2}\\ \; & =-{∥\mu ∥}^{2}+2\mu^{T}Ax-{∥Ax∥}^{2}=-{∥\mu ∥}^{2}+2x^{T}A^{T}\mu -x^{T}A^{T}Ax, \end{array}$$

where the second equality holds by the definition of μ, and the fourth equality holds by since $\sum_{i=1}^{n}x_{i}p_{i}=Ax$ for every $x\in R^{n}$. At Section 2.2 in [48], it was shown that for any quadratic function $f^{\prime }:R^{n}\to R$ that is defined as

(A5) $$f^{\prime }\left(x\right)=a+x^{T}b+x^{T}Mx,$$

where *M* is a negative semidefinite n×n matrix, $b\in R^{n}$ is a vector, and a∈R, we have that $C_{f^{\prime }}\leq diam{\left(A^{\prime }S\right)}^{2}$, where $A^{\prime }\in R^{d\times n}$ is a matrix that satisfies $M=A^{\prime T}A^{\prime }$; see equality (12) at [48]. Hence, plugging $a=-{∥\mu ∥}^{2}$, $b=2A^{T}\mu$, and $M=A^{T}A$ in (A5) yields that for the function *f* we have $C_{f}\leq diam{\left(AS\right)}^{2}$, and

$$\begin{array}{c} diam{\left(AS\right)}^{2}=\underset{a,b\in AS}{\sup}{∥a-b∥}_{2}^{2}=\underset{x,y\in S}{\sup}{∥Ax-Ay∥}_{2}^{2}. \end{array}$$

Observe that *x* and *y* are distribution vectors, thus

$$\begin{array}{c} \underset{x,y\in S}{\sup}{∥Ax-Ay∥}_{2}^{2}=\underset{i,j}{\sup}{∥p_{i}-p_{j}∥}_{2}^{2}. \end{array}$$

Since $∥p_{i}∥\leq 1$ for each i∈[n], we have that

$$\begin{array}{c} \underset{i,j}{\sup}{∥p_{i}-p_{j}∥}_{2}^{2}\leq 2. \end{array}$$

By substituting $C_{f}\leq 2$, k=8/ε, $f\left(x_{\left(k\right)}\right)=f\left(\tilde{u}\right)=-{∥\sum_{i=1}^{n}\left(w_{i}-\tilde{u_{i}}\right)p_{i}∥}^{2}$, and $f(x^{*})=0$ in (2) we get that,

(A6) $$\begin{array}{c} \frac{{∥\sum_{i=1}^{n}\left(w_{i}-\tilde{u_{i}}\right)p_{i}∥}^{2}}{8}\leq \frac{1}{8/\varepsilon +3}. \end{array}$$

Multiplying both sides of the inequality by 8 and rearranging prove Theorem (ii) as

(A7) $$\begin{array}{c} {∥\sum_{i=1}^{n}\left(w_{i}-\tilde{u_{i}}\right)p_{i}∥}^{2}\leq \frac{8}{8/\varepsilon +3}\leq \frac{8}{8/\varepsilon }=\varepsilon . \end{array}$$

**Running time:** We have $K=\left\lceil \frac{8}{\varepsilon }\right\rceil$ iterations in Algorithm 1, where each iteration takes O(nd) time, since the gradient of *f* based on the vector $x={\left(x_{1},\cdots ,x_{n}\right)}^{T}\in S$ is $-2A^{T}\sum_{i=1}^{n}\left(w_{i}-x_{i}\right)p_{i}$. This term is the multiplication between an a matrix in $R^{n\times d}$ and a vector in $R^{d}$, which takes O(nd) time. Hence, the running time of the Algorithm is $O(\frac{nd}{\varepsilon })$. □

## Appendix D. Proof of Theorem 2

**Proof of Theorem** **2.** Let (P,w) be the normalized weighted set that is computed at Lines 3–5 of Algorithm 2 where $P=\left\{p_{1},\cdots ,p_{n}\right\}$, and let $\tilde{u}=\frac{u}{{∥m∥}_{1}}$. We show that $\left(P,\tilde{u}\right)$ is a vector summarization ε-coreset for (P,w), then by Corrolary A1 we get that (Q,u) is a vector summarization ε-coreset for (Q,m). For every i∈[n] let $w_{i}^{\prime }$,$u_{i}^{\prime }$,$u_{i}$ and $p_{i}^{\prime }$ be defined as in Algorithm 2, and let $\varepsilon^{\prime }=\frac{\varepsilon }{16}$. First, by the definition of $u^{\prime }$ we have that

(A8) $$\begin{array}{c} {∥u^{\prime }∥}_{0}\leq \frac{8}{\varepsilon^{\prime }}=\frac{128}{\varepsilon }, \end{array}$$

and since $u_{i}={∥m∥}_{1}\cdot \frac{2u_{i}^{\prime }}{{∥(p_{i}^{T}|1)∥}^{2}}$ for every i∈[n], we get that

(A9) $$\begin{array}{c} {∥u∥}_{0}\leq \frac{128}{\varepsilon }. \end{array}$$

We also have by Theorem 1 that

(A10)4ε′≥4∑i=1n(wi′−ui′)p′2(A11)=4∑i=1nwi(piT∣1)2−uim1(piT∣1)22·(piT∣1)T(piT∣1)22=∑i=1n(wi−ui˜)·(piT∣1)T2(A12)=∑i=1n(wi−ui˜)·piT∣∑i=1n(wi−ui˜)T2(A13)≥∑i=1n(wi−ui˜)·pi2,

where the first derivative is by the definition of $u^{\prime }$ in Algorithm 2 at line 11, the second holds by the definition of $p^{\prime },w^{\prime }$ and *u* at Lines 8, 9, and 12 of the algorithm, the third holds since $\tilde{u}=\frac{u}{{∥m∥}_{1}}$, and the last inequality holds since ${∥(x|y)∥}^{2}\geq x^{2}$ for every $x\in R^{d}$ and y∈R. Combining the fact that $\sum_{i=1}^{n}w_{i}p_{i}=0$ with (A13) yields that

(A14) $$\begin{array}{c} 4\varepsilon^{\prime }\geq {∥\sum_{i=1}^{n}\tilde{u_{i}}p_{i}∥}^{2}. \end{array}$$

By (A12) and since *w* is a distribution vector we also have that

$$4\varepsilon^{\prime }\geq |\sum_{i=1}^{n}\left(w_{i}-\tilde{u_{i}}\right){|}^{2}={\left|1-\sum_{i=1}^{n}\tilde{u_{i}}\right|}^{2},$$

which implies

(A15) $$\;2\sqrt{\varepsilon^{\prime }}\geq \left|1-\sum_{i=1}^{n}\tilde{u_{i}}\right|.$$

Combining (A15) and (A14) yields that:

(A16) $$\begin{array}{c} {∥\frac{\sum_{i=1}^{n}\tilde{u_{i}}p_{i}}{\sum_{i=1}^{n}\tilde{u_{i}}}∥}^{2}\leq \frac{4\varepsilon^{\prime }}{{\left(1-2\sqrt{\varepsilon^{\prime }}\right)}^{2}}\leq 16\varepsilon^{\prime }=\varepsilon , \end{array}$$

where that second inequality holds since $\varepsilon^{\prime }=\varepsilon /16\leq 1/16$. By Lemma A2, Corollary A1, and (A16), Theorem 2 holds as

$${∥\sum_{i=1}^{n}\frac{u_{i}}{{∥u∥}_{1}}q_{i}-\sum_{i=1}^{n}\frac{m_{i}}{{∥m∥}_{1}}q_{i}∥}_{2}^{2}={∥\mu_{u}-\mu_{m}∥}_{2}^{2}\leq \varepsilon \sigma_{m}^{2}.$$

□

## Appendix E. Proof of Theorem 3

**Proof of Theorem** **3.** We use the notation and variable names as defined in Algorithm 3. First, we assume that w(p)>0 for every p∈P, otherwise we remove all the points in *P* which have zero weight, since they do not contribute to the weighted sum. Identify the input set $P=\left\{p_{1},\cdots ,p_{n}\right\}$ and the set *C* that is computed at Line 13 of Algorithm 3 as $C=\left\{c_{1},\cdots ,c_{\left|C\right|}\right\}$. We will first prove that the weighted set (C,u) that is computed in Lines 13–15 at an arbitrary iteration satisfies:

(a) C⊆P,
(b) $\sum_{p\in C}$*u*(*p*) =1,
(c) ${∥\sum_{p\in P}w\left(p\right)\cdot p-\sum_{p\in C}u\left(p\right)\cdot p∥}^{2}\leq \frac{\varepsilon }{\log\left(n\right)}$, and
(d) $\left|C\right|\leq \left\lceil \frac{\left|P\right|}{2}\right\rceil .$

Let $\left(\tilde{\mu },\tilde{u}\right)$ be the vector summarization $\frac{\varepsilon }{\log(n)}$-coreset of the weighted set $\left(\left\{\mu_{1},\cdots ,\mu_{k}\right\},w^{\prime }\right)$ that is computed during the execution of the current iteration at Line 12. Hence, by Theorem 1

(A17) $$\begin{array}{cc} \; & {∥\sum_{\mu_{i}\in \tilde{\mu }}\tilde{u}\left(\mu_{i}\right)\mu_{i}-\sum_{i=1}^{k}w^{\prime }\left(\mu_{i}\right)\cdot \mu_{i}∥}^{2}\leq \frac{\varepsilon }{\log(n)},\\ & \tilde{\mu }\subseteq \left\{\mu_{1},\cdots ,\mu_{k}\right\},\;\mathrm{and}\\ & |\tilde{\mu }|\leq \frac{8\cdot \log(n)}{\varepsilon }. \end{array}$$

**Proof of (a).** Property (i) is satisfied by Line 13 as we have that C⊆P. **Proof of (b).** Property (ii) is also satisfied since

(A18) $$\begin{array}{cc} \sum_{p\in C}u\left(p\right) & =\sum_{\mu_{i}\in \tilde{\mu }}\sum_{p\in P_{i}}\frac{\tilde{u}\left(\mu_{i}\right)w\left(p\right)}{w^{\prime }\left(\mu_{i}\right)}=\sum_{\mu_{i}\in \tilde{\mu }}\frac{\tilde{u}\left(\mu_{i}\right)}{w^{\prime }\left(\mu_{i}\right)}\sum_{p\in P_{i}}w(p)\\ \; & =\sum_{\mu_{i}\in \tilde{\mu }}\frac{\tilde{u}\left(\mu_{i}\right)}{\sum_{p\in P_{i}}w\left(p\right)}\sum_{p\in P_{i}}w(p)=\sum_{\mu_{i}\in \tilde{\mu }}\tilde{u}\left(\mu_{i}\right)=1, \end{array}$$

where the first equality holds by the definition of *C* at Line 13 and w(p) for every p∈C at Line 15, and the third equality holds by the definition of $u^{\prime }\left(\mu_{i}\right)$ for every $\mu_{i}\in \tilde{\mu }$ as in Line 10. **Proof of (c).** By the definition of $w^{\prime }$ and $\mu_{i}$, for every $i\in \left\{1,\cdots ,k\right\}$

(A19) $$\begin{array}{cc} \sum_{i=1}^{k}w^{\prime }\left(\mu_{i}\right)\cdot \mu_{i} & =\sum_{i=1}^{k}w^{\prime }\left(\mu_{i}\right)\cdot \left(\frac{1}{w^{\prime }\left(\mu_{i}\right)}\cdot \sum_{p\in P_{i}}w\left(p\right)\cdot p\right)\\ & =\sum_{i=1}^{k}\sum_{p\in P_{i}}w\left(p\right)p=\sum_{p\in P}w\left(p\right)p. \end{array}$$

The weighted sum of (C,u) is

(A20) $$\begin{array}{cc} \; & \sum_{p\in C}u\left(p\right)p=\sum_{\mu_{i}\in \tilde{\mu }}\sum_{p\in P_{i}}\frac{\tilde{u}\left(\mu_{i}\right)w\left(p\right)}{w^{\prime }\left(\mu_{i}\right)}\cdot p\\ & =\sum_{\mu_{i}\in \tilde{\mu }}\tilde{u}\left(\mu_{i}\right)\sum_{p\in P_{i}}\frac{w(p)}{w^{\prime }\left(\mu_{i}\right)}p=\sum_{\mu_{i}\in \tilde{\mu }}\tilde{u}\left(\mu_{i}\right)\mu_{i}, \end{array}$$

where the first equality holds by the definitions of *C* and *w*, and the third equality holds by the definition of $\mu_{i}$ at Line 9. Plugging (A19) and (A20) in (A17) satisfies (iii) as

(A21) $$\begin{array}{c} {∥\sum_{p\in P}w\left(p\right)\cdot p-\sum_{p\in C}u\left(p\right)\cdot p∥}^{2}\leq \frac{\varepsilon }{\log\left(n\right)}. \end{array}$$

**Proof of (d).** By (A17) we have that *C* contains at most $\frac{\log(n)}{\varepsilon }$ clusters from *P* and at most $\left|C\right|\leq \frac{\log(n)}{\varepsilon }\cdot \left\lceil \frac{n}{k}\right\rceil$ points, and by plugging $k=\frac{2\log(n)}{\varepsilon }$ we obtain that $\left|C\right|\leq \left\lceil \frac{\left|P\right|}{2}\right\rceil$ as required. We now prove (i)–(iii) from Theorem 3. **Proof of Theorem 3 (i).**The first condition |C|≤8/ε in (i) is satisfied since at each iteration we reduce the data size by a factor of 2, and we keep reducing until we reach the stopping condition, which is $O(\frac{\log(n)}{\varepsilon })$ by Theorem 1 (since we require a $\frac{\varepsilon }{\log(n)}$ error when we use Theorem 1, i.e., we need coreset of size $O(\frac{\log(n)}{\varepsilon })$). Then, at Line 5 when the if condition is satisfied (it should be, as explained) we finally use Theorem 1 again to obtain a coreset of size $\left\lceil 8/\varepsilon \right\rceil$ with ε-error on the small data (that was of size $\frac{O(\log(n)}{\varepsilon }$). The second condition in (i) is satisfies since at each iteration we either return such a pair (C,u) at Line 18, we get by (b) that the sum of weight is always equal to 1. **Proof of Theorem 3 (ii).** By (d) we also get that we have at most log(n) recursive calls. Hence, by induction on (2) we conclude that last computed set (C,u) at Line 18 satisfies (ii)

$${∥\sum_{p\in P}w\left(p\right)\cdot p-\sum_{p\in C}w\left(p\right)\cdot p∥}^{2}\leq \log\left(n\right)\cdot \frac{\varepsilon }{\log\left(n\right)}=\varepsilon .$$

At Line we return an ε coreset for the input weighted set (P,w) that have reached the size of $\left(\frac{\log(n)}{\varepsilon }\right)$. Hence, the output of a the call satisfies ${∥\sum_{p\in P}w\left(p\right)\cdot p-\sum_{p\in C}w\left(p\right)\cdot p∥}^{2}\leq 2\varepsilon .$ **Proof of Theorem 3 (iii).** As explained before, there are at most log(n) recursive calls before the stopping condition at Line 4 is met. At each iteration we compute the set of means $\tilde{\mu }$, and compute a vector summarization $\left(\frac{\varepsilon }{\log n}\right)$-coreset for them. Hence, the time complexity of each iteration is $n^{\prime }d+T\left(k,d,\frac{\varepsilon }{\log(n)}\right)$ where $n^{\prime }$ is the number of points in the current iteration, and $T(k,d,\frac{\varepsilon }{\log(n)})$ is the running time of Algorithm 1 on *k* points in $R^{d}$ to obtain a $\frac{\varepsilon }{\log(n)}$-coreset. Thus, the total running of time the algorithm until the "If" condition at Line 4 is satisfied is

$$\begin{array}{cc} \; & \sum_{i=1}^{\log(n)}\left(\frac{nd}{2^{i-1}}+T\left(k,d,\frac{\varepsilon }{\log(n)}\right)\right)\\ & \leq 2nd+\log\left(n\right)\cdot T\left(k,d,\frac{\varepsilon }{\log(n)}\right)\in O\left(nd+\frac{kd}{\frac{\varepsilon }{\log(n)}}\right). \end{array}$$

Plugging $k=\frac{2\log(n)}{\varepsilon }$ and observing the the last compression at Line 5 is done on a data of size $O(\frac{\log(n)}{\varepsilon })$ proves (iii) as the running time of Algorithm 3 is $O\left(nd+\frac{\log{\left(n\right)}^{2}d}{\varepsilon^{2}}\right)$. □

## Appendix F. Proof of Corollary 4

**Proof.** The corollary immediately holds by using Algorithm 2 with a small change. We change Line 11 in Algorithm 2 to use Algorithm 3 and Theorem 3, instead of Algorithm 1 and Theorem 1. □

## Appendix G. Proof of Lemma 5

We first prove the following lemma:

**Lemma** **A4.**
*Let P be a set of n points in $R^{d}$, $\mu =\frac{1}{n}\sum_{p\in P}p$, and $\sigma^{2}=\frac{1}{n}\sum_{p\in P}{∥p-\mu ∥}^{2}$. Let ε,δ∈(0,1), and let S be a sample of $m=\frac{1}{\varepsilon \delta }$ points chosen i.i.d uniformly at random from P. Then, with probability at least 1−δ we have that ${∥\frac{1}{m}\sum_{p\in S}p-\mu ∥}^{2}\leq \varepsilon \sigma^{2}.$*

**Proof.** For any random variable *X*, we denote by E(X) and var(X) the expectation and variance of the random variable *X*, respectively. Let $x_{i}$ denote the random variable that is the *i*th sample for every i∈[m]. Since the samples are drawn i.i.d, we have

(A22) $$\begin{array}{cc} \mathrm{var}\left(\frac{1}{m}\sum_{p\in S}p\right) & =\sum_{i=1}^{m}\mathrm{var}\left(\frac{x_{i}}{m}\right)=m\cdot \mathrm{var}\left(\frac{x_{1}}{m}\right)\\ \; & =m\left(\frac{\sigma^{2}}{m^{2}}\right)=\frac{\sigma^{2}}{m}=\varepsilon \delta \sigma^{2}. \end{array}$$

For any random variable *X* and error parameter $\varepsilon^{\prime }\in \left(0,1\right)$, the generalize Chebyshev’s inequality [64] reads that

(A23) $$\mathrm{Pr}\left(∥X-E(X)∥\geq \varepsilon^{\prime }\right)\leq \frac{\mathrm{var}(X)}{{\left(\varepsilon^{\prime }\right)}^{2}}.$$

Substituting $X=\frac{1}{m}\sum_{p\in S}p$, E(X)=μ and $\varepsilon^{\prime }=\sqrt{\varepsilon }\sigma$ in (A23) yields that

(A24) $$\mathrm{Pr}\left(∥\frac{1}{m}\sum_{p\in S}p-\mu ∥\geq \sqrt{\varepsilon }\sigma \right)\leq \frac{\mathrm{var}(\frac{1}{m}\sum_{p\in S}p)}{\sigma^{2}\varepsilon }.$$

Combining (A22) with (A24) proves the lemma as:

(A25) $$\mathrm{Pr}\left({∥\frac{1}{m}\sum_{p\in S}p-\mu ∥}^{2}\geq \varepsilon \sigma^{2}\right)\leq \frac{\varepsilon \delta \sigma^{2}}{\sigma^{2}\varepsilon }=\delta .$$

□

Now we prove Lemma 5

**Proof.** Let $\left\{S_{1},\cdots ,S_{k}\right\}$ be a set of *k* i.i.d sampled subsets each of size $\frac{4}{\varepsilon }$ as defined at Line 5 of Algorithm 4, and let ${\bar{s}}_{i}$ be the mean of the *i*th subset $S_{i}$ as define at Line 6. Let $\hat{s}:=\underset{x\in R^{d}}{arg min}\sum_{i=1}^{k}{∥{\bar{s}}_{i}-x∥}_{2}$ be the geometric median of the set of means $\left\{{\bar{s}}_{1},\cdots ,{\bar{s}}_{k}\right\}$. Using Corollary 4.1 from [65] we obtain that

$$\mathrm{Pr}\left(∥\hat{s}-\mu ∥\geq 11\sqrt{\frac{\sigma^{2}\log\left(1.4/\delta \right)}{\frac{4k}{\varepsilon }}}\right)\leq \delta ,$$

from the above we have that

(A26) $$\begin{array}{c} \mathrm{Pr}\left({∥\hat{s}-\mu ∥}^{2}\geq 121\frac{\varepsilon \sigma^{2}\log\left(1.4/\delta \right)}{4k}\right)\leq \delta . \end{array}$$

Note that

(A27)Prs^−μ2≥121εσ2log(1.4/δ)4k(A28)=Prs^−μ2≥30.25·εσ2log(1.4/δ)3.5log1δ+1(A29)≥Prs^−μ2≥31·εσ2,

where (A28) holds by substituting $k=3.5\log\left(\frac{1}{\delta }\right)+1$ as in Line 3 of Algorithm 4, and (A29) holds since $\frac{\log(1.4/\delta )}{3.5\log\left(\frac{1}{\delta }\right)+1}<1$ for every δ≤0.9 as we assumed. Combining (A29) with (A26) yields,

(A30) $$\begin{array}{c} \mathrm{Pr}\left({∥\hat{s}-\mu ∥}^{2}\geq 31\cdot \varepsilon \sigma^{2}\right)\leq \delta . \end{array}$$

For every i∈[k], by substituting $S=S_{i}$, which is of size $\frac{4}{\varepsilon }$, in Lemma A4, we obtain that

$$\mathrm{Pr}({∥{\bar{s}}_{i}-\mu ∥}^{2}\geq \varepsilon \sigma^{2})\leq 1/4.$$

Hence, with probability at least $1-{\left(1/4\right)}^{k}$ there is at least one set $S_{j}$ such that

$${∥{\bar{s}}_{j}-\mu ∥}^{2}\leq \varepsilon \sigma^{2}.$$

By the following inequalities:

$${\left(1/4\right)}^{k}={\left(1/4\right)}^{3.5\log\left(\frac{1}{\delta }\right)+1}\leq {\left(1/4\right)}^{\log(1/\delta )}=4^{\log(\delta )}\leq 2^{\log(\delta )}=\delta$$

we get that with probability at least 1−δ there is a set $S_{j}$ such that

(A31) $$\begin{array}{c} {∥{\bar{s}}_{j}-\mu ∥}^{2}\leq \varepsilon \sigma^{2}. \end{array}$$

Combining (A31) with (A30) yields that with probability at least ${\left(1-\delta \right)}^{2}$ the set $S_{j}$ satisfies that

(A32) $$\begin{array}{c} {∥{\bar{s}}_{j}-\hat{s}∥}^{2}\leq 32\varepsilon \sigma^{2}. \end{array}$$

Let $f:R^{d}\to \left[0,\infty \right)$ be a function such that $f\left(x\right)=\sum_{i=1}^{k}{∥{\bar{s}}_{i}-x∥}_{2}$ for every $x\in R^{d}$. Therefore, by the definitions of *f* and $\hat{s}$,

$$\hat{s}:=\underset{x\in R^{d}}{arg min}\sum_{i=1}^{k}{∥{\bar{s}}_{i}-x∥}_{2}=\underset{x\in R^{d}}{arg min}f\left(x\right).$$

Observe that *f* is a convex function since it is a sum over convex functions. By the convexity of *f*, we get that for every pair of points p,q∈P it holds that:

(A33) $$\begin{array}{c} \mathrm{if}\;f\left(q\right)\leq f\left(p\right)\;\mathrm{then}\;∥q-\hat{s}∥\leq ∥p-\hat{s}∥. \end{array}$$

Therefore, by the definition of $i^{*}$ at in Algorithm 4 we get that

(A34) $$\begin{array}{c} i^{*}\in \underset{i\in [k]}{arg min}∥{\bar{s}}_{i}-\hat{s}∥. \end{array}$$

Now by combining (A32) with (A34) we have that:

(A35) $$\begin{array}{c} \mathrm{Pr}\left({∥{\bar{s}}_{i^{*}}-\hat{s}∥}^{2}\leq 32\varepsilon \sigma^{2}\right)\geq {\left(1-\delta \right)}^{2}. \end{array}$$

Combining (A35) with (A30) and noticing the following inequality

$$\begin{array}{cc} {\left(1-\delta \right)}^{3} & =\left(1-2\delta +\delta^{2}\right)\left(1-\delta \right)\geq \left(1-2\delta \right)\left(1-\delta \right)\\ & =1-\delta -2\delta +2\delta^{2}\geq 1-3\delta , \end{array}$$

satisfies Lemma 5 as,

$$\mathrm{Pr}\left({∥{\bar{s}}_{i^{*}}-\mu ∥}^{2}\leq 33\varepsilon \sigma^{2}\right)\leq 1-3\delta .$$

**Running time.** It takes $O\left(\frac{d\log\left(\frac{1}{\delta }\right)}{\varepsilon }\right)$ to compute the set of means at Line 6, and $O\left(d\log{\left(\frac{1}{\delta }\right)}^{2}\right)$ time to compute Line 7 by simple exhaustive search over all the means. Hence, the total running time is $O\left(d\left(\log{\left(\frac{1}{\delta }\right)}^{2}+\frac{\log\left(\frac{1}{\delta }\right)}{\varepsilon }\right)\right)$. □

## Appendix H. Proof of Theorem 6

We first show a reduction to a normalized weighted set as follows:

**Corollary** **A5.**
*Let (Q,m) be a weighted set, and let (P,w) be its corresponding normalized weighted set as computed in Observation A1. Let (P,u) be a 1-mean ε-coreset for (P,w) and let $u^{\prime }={∥m∥}_{1}\cdot u$. Then $\left(Q,u^{\prime }\right)$ is a 1-mean ε-coreset for (Q,m).*

**Proof.** Let (P,u) be a 1-mean ε-coreset for (P,w). We prove that $\left(Q,u^{\prime }\right)$ is a 1-mean ε-coreset for (Q,m). Observe that

(A36) $$\begin{array}{cc} |\sum_{i=1}^{n}\left(m_{i}-u_{i}^{\prime }\right){∥q_{i}-x∥}^{2}| & =|\sum_{i=1}^{n}\left(m_{i}-u_{i}^{\prime }\right)\sigma^{2}{∥p_{i}-y∥}^{2}| \end{array}$$

(A37) $$\begin{array}{cc} & =|\sum_{i=1}^{n}{∥m∥}_{1}\sigma^{2}\left(w_{i}-u_{i}\right){∥p_{i}-y∥}^{2}|, \end{array}$$

where the first equality holds by (A1), and the second holds by the definition of *w* and $u^{\prime }$. Since (P,u) is a 1-mean ε-coreset for (P,w)

(A38) $$\begin{array}{cc} \; & |\sum_{i=1}^{n}{∥m∥}_{1}\sigma^{2}\left(w_{i}-u_{i}\right){∥p_{i}-y∥}^{2}|\\ \; & \leq \varepsilon \sum_{i=1}^{n}{∥m∥}_{1}\sigma^{2}w_{i}{∥p_{i}-y∥}^{2}\\ \; & =\varepsilon \sum_{i=1}^{n}m_{i}{∥q_{i}-x∥}^{2}, \end{array}$$

where the equality holds by (A1) and since $w=\frac{m}{{∥m∥}_{1}}$. The proof concludes by combining (A36) and (A38) as $|\sum_{i=1}^{n}\left(m_{i}-u_{i}^{\prime }\right){∥q_{i}-x∥}^{2}|\leq \varepsilon \sum_{i=1}^{n}m_{i}{∥q_{i}-x∥}^{2}.$ □

### 1-Mean Problem Reduction

Given a normalized weighted set (P,w) as in Definition A1, in the following lemma we prove that a weighted set (P,u) is a 1-mean ε-coreset for (P,w) if some three properties related to the mean, variance, and weights of (P,u) hold.

**Lemma** **A6.**
*Let (P,w) be a normalized weighted set of n points in $R^{d}$, ε∈(0,1), and $u\in R^{n}$ such that,*

1. *$∥\sum_{i=1}^{n}u_{i}p_{i}∥\leq \varepsilon$,*
2. *$|1-\sum_{i=1}^{n}u_{i}|\leq \varepsilon$, and*
3. *$|1-\sum_{i=1}^{n}u_{i}\cdot {∥p_{i}∥}^{2}|\leq \varepsilon$.*

*Then, (P,u) is a 1-mean ε-coreset for (P,w), i.e., for every $x\in R^{d}$ we have that*

(A39)
$$|\sum_{i=1}^{n}\left(w_{i}-u_{i}\right){∥p_{i}-x∥}^{2}|\leq 2\varepsilon \sum_{i=1}^{n}w_{i}{∥p_{i}-x∥}^{2}.$$

**Proof.** First we have that,

(A40) $$\begin{array}{cc} \sum_{i=1}^{n}w_{i}{∥p_{i}-x∥}^{2} & =\sum_{i=1}^{n}w_{i}{∥p_{i}∥}^{2}-2x^{T}\sum_{i=1}^{n}w_{i}p_{i}+{∥x∥}^{2}\sum_{i=1}^{n}w_{i} \end{array}$$

(A41) $$\begin{array}{cc} \; & =1+{∥x∥}^{2}, \end{array}$$

where the last equality holds by the attributes (a)–(c) of the normalized weighted set (P,w). By rearranging the left hand side of (A39) we get,

(A42)∑i=1n(wi−ui)pi−x2=∑i=1n(wi−ui)(pi2−2piTx+x2)(A43)≤∑i=1n(wi−ui)pi2+x2∑i=1n(wi−ui)+2xT∑i=1n(wi−ui)pi(A44)=1−∑i=1nuipi2+x21−∑i=1nui+2xT∑i=1nuipi(A45)≤ε+εx2+2x∑i=1nuipi,

where (A43) holds by the triangle inequality, (A44) holds by attributes (a)–(c), and (A45) holds by combining assumptions (2), (3), and the Cauchy-Schwarz inequality, respectively. We also have for every a,b≥0 that $2ab\leq a^{2}+b^{2}$, hence,

(A46) $$\begin{array}{c} 2ab=2\sqrt{\varepsilon }a\frac{b}{\sqrt{\varepsilon }}\leq \varepsilon a^{2}+\frac{b^{2}}{\varepsilon }. \end{array}$$

By (A46) and assumption (1) we get that,

(A47) $$\begin{array}{cc} 2∥x∥∥\sum_{i=1}^{n}u_{i}p_{i}∥\leq & \varepsilon {∥x∥}^{2}+\frac{{∥\sum_{i=1}^{n}u_{i}p_{i}∥}^{2}}{\varepsilon }\\ \; & \leq \varepsilon {∥x∥}^{2}+\frac{\varepsilon^{2}}{\varepsilon }\\ \; & =\varepsilon {∥x∥}^{2}+\varepsilon . \end{array}$$

Lemma A6 now holds by plugging (A47) in (A45) as,

(A48) $$\begin{array}{cc} \left|\sum_{i=1}^{n}\left(w_{i}-u_{i}\right){∥p_{i}-x∥}^{2}\right| & \leq \varepsilon +\varepsilon {∥x∥}^{2}+\varepsilon {∥x∥}^{2}+\varepsilon \\ \; & =2\varepsilon +2\varepsilon {∥x∥}^{2}\\ \; & =2\varepsilon (1+{∥x∥}^{2})\\ \; & =2\varepsilon \sum_{i=1}^{n}w_{i}{∥p_{i}-x∥}^{2}, \end{array}$$

where the last equality holds by (A41). Observe that if assumptions (1), (2) and (3) hold, then (A48) hold. We therefore obtain an ε-coreset. □

To Proof Theorem 6, we split it into 2 claims:

**Claim** **A7.**
*Let (Q,m) be a weighted set of n points in $R^{d}$, ε∈(0,1), and let u be the output of a call to $CoreSet(Q,m,{\left(\frac{\varepsilon }{4}\right)}^{2})$; see Algorithm 2. Then $u=\left(u_{1},\cdots ,u_{n}\right)\in R^{n}$ is a vector with ${∥u∥}_{0}\leq \frac{128}{\varepsilon^{2}}$ non-zero entries that is computed in $O(\frac{nd}{\varepsilon^{2}})$ time, and (Q,u) is a 1-mean ε-coreset for (Q,m).*

**Proof.** Let (P,w) be the normalized weighted set that is computed at Lines 3–5 of Algorithm 2 where $P=\left\{p_{1},\cdots ,p_{n}\right\}$, and let $\tilde{u}=\frac{u}{{∥m∥}_{1}}$. We show that $\left(P,\tilde{u}\right)$ is a 1-mean ε-coreset for (P,w), then by Corollary A5 we get that (Q,u) is a 1-mean coreset for (Q,m). Let $\varepsilon^{\prime }=\frac{\varepsilon }{4}$, let $p_{i}^{\prime }:=\frac{{\left(p_{i}^{T}|1\right)}^{T}}{{∥(p_{i}^{T}|1)∥}^{2}}$ and $w_{i}^{\prime }:=\frac{w_{i}{∥(p_{i}^{T}|1)∥}^{2}}{2}$ for every i∈[n]. By the definition of $u^{\prime }$ at line 11 in Algorithm 2, and since the algorithm gets ${\varepsilon^{\prime }}^{2}$ as input, we have that

(A49) $$\begin{array}{c} {∥u^{\prime }∥}_{0}\leq 8/{\varepsilon^{\prime }}^{2}=\frac{128}{\varepsilon^{2}}, \end{array}$$

and

(A50) $$\begin{array}{c} {∥\sum_{i=1}^{n}\left(w_{i}^{\prime }-u_{i}^{\prime }\right)p_{i}^{\prime }∥}^{2}\leq {\varepsilon^{\prime }}^{2}. \end{array}$$

For every i∈[n] let $u_{i}={∥m∥}_{1}\cdot \frac{2u_{i}^{\prime }}{{∥(p_{i}^{T}|1)∥}^{2}}$ be defined as at Line 12 of the algorithm. It immediately follows by the definition of $u=(u_{1},\cdots ,u_{n})$ and (A49) that

(A51) $$\begin{array}{c} {∥u∥}_{0}\leq 128/{\varepsilon^{\prime }}^{2}. \end{array}$$

We now prove that Properties (1)–(3) in Lemma A6 hold for $\left(P,\tilde{u}\right)$. We have that

(A52)2ε′≥2∑i=1n(wi′−ui′)pi′=2∑i=1nwi(piT∣1)2−uim1(piT∣1)22·(piT∣1)T(piT∣1)2(A53)=∑i=1n(wi−ui˜)·(piT∣1)T(A54)=∑i=1n(wi−ui˜)·piT∣∑i=1n(wi−ui˜)T(A55)≥∑i=1n(wi−ui˜)·pi,

where the first derivation follows from (A50), the second holds by the definition of $w_{i}^{\prime }$,$u_{i}^{\prime }$,$u_{i}$ and $p_{i}^{\prime }$ for every i∈[n], the third holds since $\tilde{u}=\frac{u}{{∥m∥}_{1}}$, and the last holds since $∥(x|y)∥\geq ∥x∥$ for every x,y such that $x\in R^{d}$ and y∈R. By (A54) and since *w* is a distribution vector we also have that

(A56) $$\begin{array}{c} 2\varepsilon^{\prime }\geq |\sum_{i=1}^{n}\left(w_{i}-\tilde{u_{i}}\right)|=\left|1-\sum_{i=1}^{n}\tilde{u_{i}}\right|. \end{array}$$

By Theorem 1, we have that $u^{\prime }$ is a distribution vector, which yields,

$$\begin{array}{c} 2=2\sum_{i=1}^{n}u_{i}^{\prime }=\sum_{i=1}^{n}\tilde{u_{i}}{∥{\left(p_{i}^{T}|1\right)}^{T}∥}^{2}=\sum_{i=1}^{n}\tilde{u_{i}}{∥p_{i}∥}^{2}+\sum_{i=1}^{n}\tilde{u_{i}}, \end{array}$$

By the above we get that $2-\sum_{i=1}^{n}\tilde{u_{i}}=\sum_{i=1}^{n}\tilde{u_{i}}{∥p_{i}∥}^{2}$. Hence,

(A57) $$\begin{array}{cc} \; & |\sum_{i=1}^{n}\left(w_{i}-\tilde{u_{i}}\right){∥p_{i}∥}^{2}\left|=\right|\sum_{i=1}^{n}w_{i}{∥p_{i}∥}^{2}-\left(2-\sum_{i=1}^{n}\tilde{u_{i}}\right)|\\ & =|1-(2-\sum_{i=1}^{n}\tilde{u_{i}})\left|=\right|\sum_{i=1}^{n}\tilde{u_{i}}-1|\leq 2\varepsilon^{\prime } \end{array}$$

where the first equality holds since $\sum_{i=1}^{n}\tilde{u_{i}}{∥p_{i}∥}^{2}=2-\sum_{i=1}^{n}\tilde{u_{i}}$, the second holds since *w* is a distribution and the last is by (A56). Now by (A57), (A56) and (A55) we obtain that $\left(P,\tilde{u_{i}}\right)$ satisfies Properties (1)–(3) in Lemma A6. Hence, by Lemma A6 and Corollary A5 we get that

$$\begin{array}{cc} |\sum_{i=1}^{n}\left(w_{i}-u_{i}\right){∥p_{i}-x∥}^{2}| & \leq 4\varepsilon^{\prime }\sum_{i=1}^{n}w_{i}{∥p_{i}-x∥}^{2}\\ & =\varepsilon \sum_{i=1}^{n}w_{i}{∥p_{i}-x∥}^{2}. \end{array}$$

The running time is the running time of Algorithm 1 with $\varepsilon^{2}$ instead of ε, i.e., $O(nd/\varepsilon^{2})$. □

Now we proof the following claim:

**Claim** **A8.**
*Let (Q,m) be a weighted set of n points in $R^{d}$, ε∈(0,1). Then in $O(nd+d\cdot \frac{\log{\left(n\right)}^{2}}{\varepsilon^{4}})$ we can compute a vector $u={\left(u_{1},\cdots ,u_{n}\right)}^{T}\in R^{n}$, such that u has ${∥u∥}_{0}\leq \frac{128}{\varepsilon^{2}}$ non-zero entries, and (Q,u) is a 1-mean (2ε)-coreset for (Q,m).*

**Proof.** The Claim immediately holds by using Algorithm 2 with a small change. We change Line 11 in Algorithm 2 to use Algorithm 3 and Theorem 3, instead of Algorithm 1 and Theorem 1. □

Combining both Claim A7 with Claim A8 proves Theorem 6.

## Appendix I. Proof of Corollary 7

**Proof.** We consider the variables defined in Algorithm 5. Let $X\in R^{d\times (d-k)}$ such that $X^{T}X=I$, and let $A^{\prime }=\left[A|{\left(r,\cdots ,r\right)}^{T}\right]$. Plugging $A=A^{\prime }$ into Theorem 3 at [35]

(A58) $$|1-\frac{{∥WA^{\prime }X∥}^{2}}{{∥A^{\prime }X∥}^{2}}|\leq 5∥\sum_{i=1}^{n}\tilde{v_{i}}-W_{i,i}^{2}\tilde{v_{i}}∥.$$

We also have by the definition of *W* and Theorem 2

(A59) $$\begin{array}{c} ∥\sum_{i=1}^{n}\tilde{v_{i}}-W_{i,i}^{2}\tilde{v_{i}}∥\leq \left(\varepsilon /k\right)\sqrt{\sum_{i=1}^{n}{∥\tilde{v_{i}}∥}^{2}}\leq \left(\varepsilon /k\right)\sum_{i=1}^{n}∥\tilde{v_{i}}∥, \end{array}$$

where the first inequality holds since $W_{i,i}=u_{i}^{2}$ for every i∈[n], and the vector $u\in R^{n}$ is a vector summarization ${\left(\varepsilon /5k\right)}^{2}$-coreset for $\left(\left\{\tilde{v_{1}},\cdots ,\tilde{v_{n}}\right\},\left(1,\cdots ,1\right)\right)$. Finally, at [35] they show that $\left(\varepsilon /5k\right)\sum_{i=1}^{n}∥\tilde{v_{i}}∥\leq \varepsilon$. Hence, combining this fact with (A58), and (A59) yields

(A60) $$\begin{array}{c} |1-\frac{{∥WA^{\prime }X∥}^{2}}{{∥A^{\prime }X∥}^{2}}|\leq \varepsilon . \end{array}$$

Finally, the corollary holds by combing Lemma 4.1 at [45] with (A60). □

## References

[1] Valiant L.G. (1984). A theory of the learnable. Commun. ACM.

[2] Vapnik V. (1992). Principles of risk minimization for learning theory. Advances in Neural Information Processing Systems.

[3] Feldman D., Langberg M. A unified framework for approximating and clustering data. Proceedings of the Forty-Third Annual ACM Symposium on Theory of Computing.

[4] Nielsen M.A. (2015). Neural Networks and Deep Learning.

[5] Steinwart I., Christmann A. (2008). Support Vector Machines.

[6] Tibshirani R. (1996). Regression shrinkage and selection via the lasso. J. R. Stat. Soc. Ser. B (Methodol.).

[7] Hastie T., Tibshirani R., Friedman J. (2009). The Elements of Statistical Learning: Data Mining, Inference, and Prediction.

[8] Hoerl A.E., Kennard R.W. (1970). Ridge regression: Biased estimation for nonorthogonal problems. Technometrics.

[9] Bergman S. (1970). The Kernel Function and Conformal Mapping.

[10] Eggleston H.G. (1966). Convexity. J. Lond. Math. Soc..

[11] Phillips J.M. (2016). Coresets and sketches. arXiv.

[12] Har-Peled S. (2011). Geometric Approximation Algorithms.

[13] Vapnik V. (2013). The Nature of Statistical Learning Theory.

[14] Langberg M., Schulman L.J. Universal *ε*-approximators for integrals. Proceedings of the Twenty-First Annual ACM-SIAM Symposium on Discrete Algorithms.

[15] Carathéodory C. (1907). Über den Variabilitätsbereich der Koeffizienten von Potenzreihen, die gegebene Werte nicht annehmen. Math. Ann..

[16] Cook W., Webster R. (1972). Caratheodory’s theorem. Can. Math. Bull..

[17] Phillips J.M., Tai W.M. (2020). Near-optimal coresets of kernel density estimates. Discret. Comput. Geom..

[18] Matousek J. (1995). Approximations and optimal geometric divide-and-conquer. J. Comput. Syst. Sci..

[19] Braverman V., Feldman D., Lang H. (2016). New frameworks for offline and streaming coreset constructions. arXiv.

[20] Bentley J.L., Saxe J.B. (1980). Decomposable searching problems I: Static-to-dynamic transformation. J. Algorithms.

[21] Har-Peled S., Mazumdar S. On coresets for k-means and k-median clustering. Proceedings of the Thirty-Sixth Annual ACM Symposium on Theory of Computing.

[22] Maalouf A., Jubran I., Feldman D. (2019). Fast and accurate least-mean-squares solvers. arXiv.

[23] Drineas P., Magdon-Ismail M., Mahoney M.W., Woodruff D.P. (2012). Fast approximation of matrix coherence and statistical leverage. J. Mach. Learn. Res..

[24] Cohen M.B., Peng R. Lp row sampling by lewis weights. Proceedings of the Forty-Seventh Annual ACM Symposium on Theory of Computing.

[25] Ritter K. (2007). Average-Case Analysis of Numerical Problems.

[26] Juditsky A., Nemirovski A.S. (2008). Large deviations of vector-valued martingales in 2-smooth normed spaces. arXiv.

[27] Tropp J.A. (2015). An introduction to matrix concentration inequalities. arXiv.

[28] Charikar M., Chen K., Farach-Colton M. (2002). Finding frequent items in data streams. International Colloquium on Automata, Languages, and Programming.

[29] Feldman D., Ozer S., Rus D. Coresets for vector summarization with applications to network graphs. Proceedings of the 34th International Conference on Machine Learning.

[30] Węglarczyk S. (2018). Kernel density estimation and its application. ITM Web of Conferences.

[31] Zheng Y., Jestes J., Phillips J.M., Li F. Quality and efficiency for kernel density estimates in large data. Proceedings of the 2013 ACM SIGMOD International Conference on Management of Data.

[32] Bachem O., Lucic M., Krause A. Scalable k-means clustering via lightweight coresets. Proceedings of the 24th ACM SIGKDD International Conference on Knowledge Discovery & Data Mining.

[33] Barger A., Feldman D. k-Means for Streaming and Distributed Big Sparse Data. Proceedings of the 2016 SIAM International Conference on Data Mining.

[34] Feldman D., Schmidt M., Sohler C. (2018). Turning Big data into tiny data: Constant-size coresets for k-means, PCA and projective clustering. arXiv.

[35] Feldman D., Volkov M., Rus D. (2016). Dimensionality reduction of massive sparse datasets using coresets. Adv. Neural Inf. Process. Syst..

[36] Cohen M.B., Elder S., Musco C., Musco C., Persu M. Dimensionality reduction for k-means clustering and low rank approximation. Proceedings of the Forty-Seventh Annual ACM on Symposium on Theory of Computing.

[37] Varadarajan K., Xiao X. (2012). On the sensitivity of shape fitting problems. arXiv.

[38] Feldman D., Tassa T. More constraints, smaller coresets: Constrained matrix approximation of sparse big data. Proceedings of the 21th ACM SIGKDD International Conference on Knowledge Discovery and Data Mining.

[39] Frieze A., Kannan R., Vempala S. (2004). Fast Monte-Carlo algorithms for finding low-rank approximations. J. ACM (JACM).

[40] Yang J., Chow Y.L., Ré C., Mahoney M.W. (2017). Weighted SGD for *ℓ*_p_ regression with randomized preconditioning. J. Mach. Learn. Res..

[41] Cohen M.B., Lee Y.T., Musco C., Musco C., Peng R., Sidford A. Uniform sampling for matrix approximation. Proceedings of the 2015 Conference on Innovations in Theoretical Computer Science.

[42] Papailiopoulos D., Kyrillidis A., Boutsidis C. Provable deterministic leverage score sampling. Proceedings of the 20th ACM SIGKDD iInternational Conference on Knowledge Discovery and Data Mining.

[43] Drineas P., Mahoney M.W., Muthukrishnan S. (2008). Relative-error CUR matrix decompositions. SIAM J. Matrix Anal. Appl..

[44] Cohen M.B., Musco C., Musco C. Input sparsity time low-rank approximation via ridge leverage score sampling. Proceedings of the Twenty-Eighth Annual ACM-SIAM Symposium on Discrete Algorithms.

[45] Maalouf A., Statman A., Feldman D. Tight sensitivity bounds for smaller coresets. Proceedings of the 26th ACM SIGKDD International Conference on Knowledge Discovery & Data Mining.

[46] Batson J., Spielman D.A., Srivastava N. (2012). Twice-ramanujan sparsifiers. SIAM J. Comput..

[47] Cohen M.B., Nelson J., Woodruff D.P. (2015). Optimal approximate matrix product in terms of stable rank. arXiv.

[48] Clarkson K.L. (2010). Coresets, sparse greedy approximation, and the Frank-Wolfe algorithm. ACM Trans. Algorithms (TALG).

[49] Desai A., Ghashami M., Phillips J.M. (2016). Improved practical matrix sketching with guarantees. IEEE Trans. Knowl. Data Eng..

[50] Madariaga D., Madariaga J., Bustos-Jiménez J., Bustos B. (2021). Improving Signal-Strength Aggregation for Mobile Crowdsourcing Scenarios. Sensors.

[51] Mahendran N., Vincent D.R., Srinivasan K., Chang C.Y., Garg A., Gao L., Reina D.G. (2019). Sensor-assisted weighted average ensemble model for detecting major depressive disorder. Sensors.

[52] Wu L., Xu Q., Heikkilä J., Zhao Z., Liu L., Niu Y. (2019). A star sensor on-orbit calibration method based on singular value decomposition. Sensors.

[53] Yang W., Hong J.Y., Kim J.Y., Paik S.h., Lee S.H., Park J.S., Lee G., Kim B.M., Jung Y.J. (2020). A novel singular value decomposition-based denoising method in 4-dimensional computed tomography of the brain in stroke patients with statistical evaluation. Sensors.

[54] Peri E., Xu L., Ciccarelli C., Vandenbussche N.L., Xu H., Long X., Overeem S., van Dijk J.P., Mischi M. (2021). Singular value decomposition for removal of cardiac interference from trunk electromyogram. Sensors.

[55] (2021). Code. Open Source Code for All the Algorithms Presented in This Paper. https://github.com/alaamaalouf/vector-summarization-coreset.

[56] Van Rossum G., Drake F.L. (2009). Python 3 Reference Manual.

[57] Oliphant T.E. (2006). A Guide to NumPy.

[58] Tremblay N., Barthelmé S., Amblard P.O. (2019). Determinantal Point Processes for Coresets. J. Mach. Learn. Res..

[59] Dua D., Graff C. (2017). UCI Machine Learning Repository. http://archive.ics.uci.edu/ml.

[60] Donovan B., Work D. (2015). Using Coarse GPS Data to Quantify City-Scale Transportation System Resilience to Extreme Events. http://vis.cs.kent.edu/DL/Data/.

[61] US Census Data (1990) Data Set. https://archive.ics.uci.edu/ml/datasets/US+Census+Data+(1990).

[62] Kawala F., Douzal-Chouakria A., Gaussier E., Dimert E. (2013). Prédictions D’activité dans les Réseaux Sociaux en Ligne. https://archive.ics.uci.edu/ml/datasets/Buzz+in+social+media+.

[63] Huerta R., Mosqueiro T., Fonollosa J., Rulkov N.F., Rodriguez-Lujan I. (2016). Online decorrelation of humidity and temperature in chemical sensors for continuous monitoring. Chemom. Intell. Lab. Syst..

[64] Chen X. (2007). A new generalization of Chebyshev inequality for random vectors. arXiv.

[65] Minsker S. (2015). Geometric median and robust estimation in Banach spaces. Bernoulli.

